# Serious mental health conditions and exposure to adulthood trauma in low- and middle-income countries: a scoping review

**DOI:** 10.1017/gmh.2024.123

**Published:** 2024-11-20

**Authors:** Anne Stevenson, Engida Girma, Benon Kabale Kitafuna, Boniface Harerimana, Karestan C. Koenen, Soraya Seedat

**Affiliations:** 1 Department of Psychiatry, Faculty of Medicine and Health Sciences, Stellenbosch University, Cape Town, South Africa; 2 Department of Epidemiology, Harvard T. H. Chan School of Public Health, Boston, MA, USA; 3 Department of Psychiatry, School of Medicine, College of Health Sciences, Addis Ababa University, Addis Ababa, Ethiopia; 4 Community Mental Health Initiative NGO, Mulago Hospital Complex, Kampala, Uganda; 5 Lawson Health Research Institute, London, Ontario, Canada; 6 Department of Social and Behavioral Sciences, Harvard T.H. Chan School of Public Health, Boston, MA, USA; 7 Psychiatric & Neurodevelopmental Genetics Unit, Department of Psychiatry, Massachusetts General Hospital, Boston, MA, USA; 8 South African Medical Research Council Unit on the Genomics of Brain Disorders, Department of Psychiatry, Faculty of Medicine and Health Sciences, Stellenbosch University, Cape Town, South Africa

**Keywords:** schizophrenia, bipolar disorder, serious mental health conditions, trauma, scoping review

## Abstract

**Background:**

There is a strong link between trauma exposure and serious mental health conditions (SMHCs), such as schizophrenia and bipolar disorder. The majority of research in the field has focused on childhood trauma as a risk factor for developing an SMHC and on samples from high-income countries. There is less research on having an SMHC as a risk factor for exposure to traumatic events, and particularly on populations in low- and middle-income countries (LMICs).

This scoping review aimed to synthesize the nature and extent of research on traumatic events that adults with SMHCs face in LMICs. It was conducted across five databases: PubMed, Embase, PsycINFO, Web of Science Core Collection and Africa-Wide Information/NiPad in December 2023 and by hand searching citation lists.

**Findings:**

The database search returned 4,111 articles. After removing duplicates and following a rigorous screening process, 51 articles met criteria for inclusion. There was one case study, one mixed methods study, 12 qualitative studies and 37 quantitative studies. Ten countries were represented, with the most studies from India (n = 19), Ethiopia (n = 9) and China (n = 6). Schizophrenia was the most studied type of SMHC. Of the trauma exposures, more than 76% were on interpersonal violence, such as sexual and physical violence. Of the studies on interpersonal violence, more than 23% were on physical restraint (e.g., shackling) in the community or in hospital settings. There were no studies on man-made or natural disasters.

**Implications:**

Much of our data in this population are informed by a small subset of countries and by certain types of interpersonal violence. Future research should aim to expand to additional countries in LMICs. Additional qualitative research would likely identify and contextualize other trauma types among adults with SMHCs in LMICs.

## Graphical abstract

**Figure:**
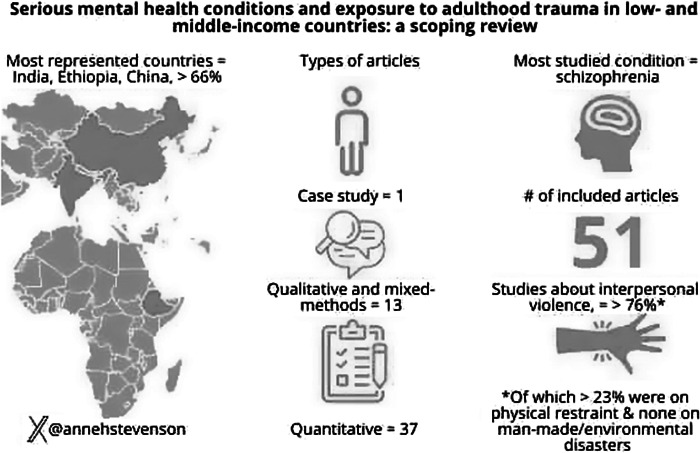

## Impact statement

The impact of this scoping review, a type of analysis that looks at all the research on a specific topic, shows much of our data on adults with preexisting serious mental health conditions (SMHCs) who are then exposed to trauma in low- and middle-income countries (LMICs) come from a small subset of countries and certain types of interpersonal violence.

History of the topic: Trauma exposure and SMHCs, such as schizophrenia and bipolar disorder, have been shown to be interconnected. Most of the research in this area has focused on childhood trauma as a risk factor for developing an SMHC, and the bulk of this work has come from studies in high-income countries in North America and Western Europe. While it is often stated that people with SMHCs are more vulnerable to experiencing traumatic events than the general public, there is less research on having an SMHC as a risk factor for exposure to traumatic events, and particularly on populations in LMICs, which have been underrepresented in research to date. This scoping review aims to fill this gap and to synthesize what trauma types adults with SMHCs face in LMICs.

Main takeaways: After a thorough search, we found that studies on this topic came from only 10 countries (out of more than 130 LMICs) and two-thirds of these were from India, Ethiopia and China. The vast majority of these studies were about interpersonal violence, such as sexual violence, intimate partner violence and physical violence, and more than one-fifth of these were on physical restraint, such as chaining. There were no studies on man-made or natural disasters. The findings support that adulthood trauma exposure among people with SMHCs is an existing issue in LMICs and is a call to action to eradicate violent practices in both community and healthcare settings.

## Introduction

There is an established link between trauma exposure and serious mental health conditions (SMHCs), such as schizophrenia, schizoaffective disorder and bipolar disorder (Grubaugh et al., 2011; Mauritz et al., 2013). Many studies have shown that trauma exposure, often defined as exposure to actual or threatened death, serious injury or sexual violence (American Psychiatric Association, 2013), poses a significant risk for the development of SMHCs (Read et al., 2005; Varese et al., 2012; Woolway et al., 2022). Most of the research on trauma and SMHCs has focused on childhood adversity and in samples from high-income countries in Europe, North America and Australasia (Read et al., 2005; Varese et al., 2012; Woolway et al., 2022).

Multiple models have been proposed to explain why exposure to trauma, and particularly childhood adversity, may increase the risk for developing an SMHC, including the stress-vulnerability model (Zubin and Spring, 1977). This model posits that some people have a biological vulnerability to SMHCs. While individuals may be able to withstand a certain amount of stress, once a certain threshold of stress is reached or a stressor is particularly intense, people become more susceptible to developing SMHCs.

Likewise, researchers have also argued that people with SMHCs may be more vulnerable to exposure to trauma than those without such conditions. Population studies have found people with SMHCs report more victimization than the general public (Maniglio, 2009; de Vries et al., 2019). One theory for this is that people with SMHCs may have cognitive impairments that impact their decision-making and problem-solving abilities, restricting their ability to navigate potentially dangerous situations (de Vries et al., 2019). People with SMHCs may also be exposed to trauma through settings due to their mental health conditions, such as inpatient and outpatient healthcare centers. Patients with SMHCs have reported high rates of trauma exposure in psychiatric settings, including physical and sexual assault by staff, police and other patients (Frueh et al., 2005; Lundberg et al., 2012a).

Like the childhood adversity literature, the research on having an SMHC as a risk factor for trauma exposure in adulthood has also primarily been from samples in high-income countries (Maniglio, 2009; de Vries et al., 2019). Deepening research in LMICs may shed light on the possibility of unique traumas in this population. Furthermore, identifying and codifying trauma exposure in this population may raise awareness about such incidents and lead to healthcare and human rights policy changes for people with SMHCs (Guan et al., 2015; Hidayat et al., 2023).

This scoping review aims to fill this gap by synthesizing the literature on trauma that adults with a diagnosis of schizophrenia, bipolar disorder, schizoaffective disorder, delusional disorder or depression with psychotic features are exposed to in LMICs, which have been less represented in research to date.

We framed this exploratory study around Peters et al.’s population, concept, context framework for scoping reviews (Peters et al., 2021). Our primary research question aimed to establish the evidence base on adult trauma exposure experienced by people with SMHCs in LMICs. In other words, what is the extent, range, and nature of research about adults with SMHCs (population) being exposed to a traumatic event in adulthood (concept) in an LMIC (context)?

Our secondary research question was to ascertain whether there were any trends in the data; for example, whether there were trauma types or demographics that were more commonly studied than others. We expected most trauma exposures would be types of interpersonal violence (use of physical, sexual or psychological force against another person or small group of people) (Mercy et al., 2017) and human rights violations, such as solitary confinement for multiple years.

## Methods

We followed the JBI updated guidelines for the conduct of scoping reviews (Peters et al., 2021). As part of the review, we developed an extensive search query, retrieved all articles, conducted multiple phases of screening, extracted the data and synthesized the results. We outline each step below.

***Search strategy:*** We developed our search strategy to run in five databases: PubMed, Embase, PsycINFO, Web of Science Core Collection and Africa-Wide Information/NiPad. We started with PubMed and used a combination of medical subject headings (MeSH), some of which were “exploded” to retrieve any citations that fell under its subheadings, title and abstract (tiab) fields, and keyword (kw) fields. We used Boolean operators to develop a search query based on more than 300 terms and phrases connected to SMHCs, traumatic events and LMICs.

For SMHCs, we included words that were often associated with this term, such as “schizophrenia,” “serious mental illness” and “psychotic disorder.”

For trauma exposure, we pulled terms from two commonly used measures that assess potentially traumatic events: the Life Events Checklist for the *Diagnostic and Statistical Manual of Mental Disorders (DSM)*, Fifth Edition (LEC-5) (Weathers et al., 2013) and the posttraumatic stress disorder module of the World Mental Health Survey version of the World Health Organization Composite International Diagnostic Interview (CIDI) (Kessler and Üstün, 2004), which is based on the International Classification of Diseases (ICD). We then expanded the terms from the LEC-5 and the CIDI to include specific examples of the type of trauma in question. For example, for “natural disasters,” we listed this term and also listed typhoons, landslides, tsunamis, etc. A priori, on the basis of qualitative studies with people with SMHCs in LMICs and commentaries by leaders in the field (Alem, 2000; Ametaj et al., 2021), we included events that may be more common in LMICs than in high-income countries, although they can happen anywhere, including human rights violations, mob justice, chaining and restraining. Additionally, we included the term “idiom(s) of distress” to potentially retrieve articles that might have focused on expressions of trauma not typically captured in “Western” terminology.

For LMICs, we included the name of every country that was classified as an LMIC by the World Bank as of November 2023 (World Bank, 2023) (i.e., Afghanistan) as well as unrecognized countries or disputed territories that were not on the World Bank list but still met criteria as an LMIC (i.e., Somaliland). We also added overarching categories that are sometimes used to describe these groupings, such as “South Asia,” “developing country,” and “Global South.”

We then adapted the search string to Embase, PsycINFO, Web of Science, and Africa-Wide Information/NiPad using their controlled subject vocabulary. See Supplemental File 1 for the full search strings for each database.

***Inclusion and exclusion criteria:*** Our inclusion and exclusion criteria were as follows:

Inclusion:

- Adults aged ≥18 years.
- People with a diagnosis of schizophrenia, bipolar disorder, schizoaffective disorder, delusional disorder or depression with psychotic features.
- Trauma exposure must have occurred after the psychiatric diagnosis.
- Study population was in an LMIC.
- Observational studies only.
- Empirical studies in peer-reviewed journals, including case studies, qualitative studies and quantitative studies.
- There were no restrictions on the language in which the article was written.
- There were no restrictions on the date the article was published.

Exclusion:

- Studies with data collected in high-income countries.
- Studies where it was not clear when the trauma occurred and the diagnosis of the mental health condition occurred.
- Studies where the trauma only occurred in childhood, adolescence or youth.
- Children (people aged <18 years).
- Intervention studies (i.e., randomized control trials).
- Conference papers and abstracts, commentaries, letters, news articles, scoping reviews, systematic reviews, meta-analyses and gray literature.
- Biological process studies such as biomarkers, genetic studies, biospecimens and circuitry.
- Animal studies.

There were a few caveats to these criteria. If we could not distinguish whether an article included participants with depression vs. depression with psychotic features, we kept the article in. If an article was about “psychiatric conditions,” but we could not split out data from the disorders that met our eligibility criteria (i.e., schizophrenia) from the overall grouping, we excluded the article. If an article included participants under 17.9 years and we could separate out data for participants ≥18, we included the article and only reported on data pertaining to adults. If we could not split out participants under 17.9 from ≥18 (for example, if the age range was 15–49), the article met all our other eligibility criteria and the mean age of participants was ≥18, we included the article.

### Reviewing retrieved articles

**Figure 1.**
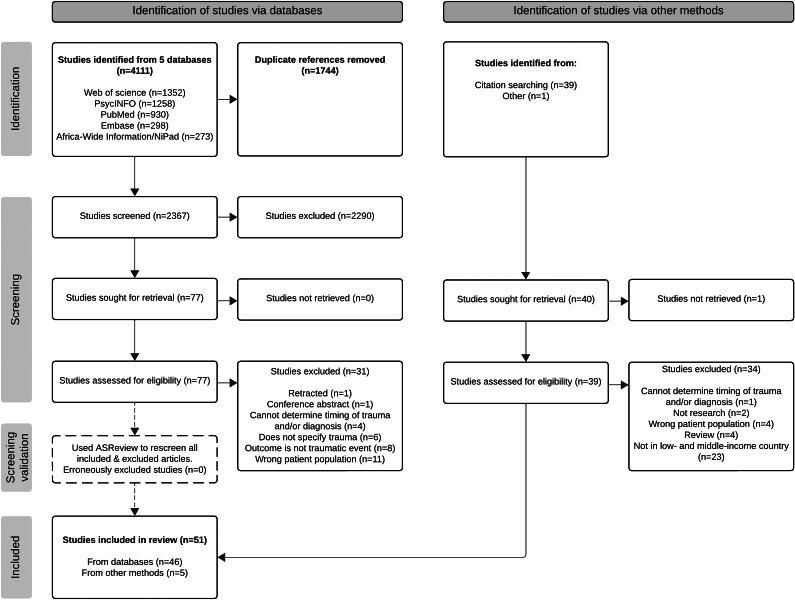
PRISMA flow chart of the search and selection process for the scoping review.

We ran our search on December 20, 2023, downloaded the citations from the retrieved articles and imported them into Covidence (www.covidence.org [Veritas Health Innovation Ltd, 2024]). Following the removal of duplicate articles in Covidence, we followed Preferred Reporting Items for Systematic Reviews and Meta-Analyses Extension for Scoping Reviews (PRISMA-ScR) (Tricco et al., 2018) process and created a flowchart to capture each stage (see Figure 1).

We screened the remaining articles in two stages. First, two reviewers (AS and EG) independently screened the titles and abstracts for eligibility according to the inclusion/exclusion criteria. The same reviewers then independently screened the full text of the remaining articles to assess eligibility. At both stages, for articles that were in conflict, the two reviewers discussed the articles and came to a consensus on whether they met eligibility. Following confirmation that an article met the criteria, we hand-searched its reference list to identify additional articles that should be reviewed (Dundar and Fleeman, 2017).

We then moved all articles into the data extraction pool. We created a Google Sheet to extract a range of fields from each article: article information (i.e., first author and title), study design, sample size, sex/gender of participants (% female), mean age, racial/ethnic composition, participant psychiatric diagnosis, method of assessment for the psychiatric condition, type of trauma exposure, tool used to assess trauma exposure, and country and municipality where the participants were recruited. AS extracted data for each article, and another reviewer independently extracted data from the same article. They then cross-checked each other’s work to ensure accuracy. We held group discussions when there were divergent decisions and came to a consensus on the final answer.

### Search procedure validation

In order to validate our search procedure and to confirm that we did not exclude any articles in the screening stages that should have been included, we used a machine learning tool, ASReview version 1.6.2 (https://asreview.nl/ [van de Schoot et al., 2021]) to additionally re-check the excluded articles. ASReview is an open-source software that utilizes active-learning to screen large amounts of data primarily for scoping and systematic reviews. For this process, we uploaded all the articles from the original screening phase (following the removal of the duplicates). We then selected one article that met inclusion criteria and one that did not train the software on which articles should be included/excluded. We screened 407 articles in total, labeling each one as “relevant” (likely met inclusion criteria) and “irrelevant” (did not meet inclusion criteria). From the articles we labeled as relevant, we cross-checked these against our final list of included articles and reread the full text of any articles we had originally excluded to determine whether they met inclusion criteria.

### Synthesis plan

The synthesis plan was a narrative summary following guidelines from (Cumpston et al., 2022) in which we focused primarily on the types of studies included, countries where the data collection took place and types of trauma reported. We presented the charting results as text in a table and graphical summaries of the research data.

We did not conduct a formal publication bias analysis as this is not typically done in scoping reviews (Peters et al., 2021).

### Protocol deposit

The protocol for this study is available through Open Science Framework and can be found here: https://doi.org/10.17605/OSF.IO/JRSQP.

## Results

We ran our search in PubMed, Embase, PsycINFO, Web of Science Core Collection and Africa-Wide Information/NiPad, which retrieved 4,111 articles. After uploading all search results into Covidence, we removed 1,744 duplicates and were left with 2,367 articles for title and abstract screening. AS and EG double screened all titles and abstracts and removed 2,290 irrelevant studies. Their agreement rate was 97.5%. We then reviewed the full text of 77 articles. Forty-six studies met the criteria for inclusion. For the hand-searching process, AS identified 39 citations for articles to screen; after going through the full screening process for these articles, AS found four that met eligibility. In addition, AS discovered an additional article that met the criteria during a separate literature review. AS added these five articles to the pool for extraction. Using ASReview, AS tagged 57 articles as relevant, of which 46 were already in our included pool. Upon rereading the additional 11, they still did not meet our inclusion criteria; thus, the software did not identify any erroneously excluded articles.

We were left with 51 articles that met the criteria. (See Figure 1 for the PRISMA flow diagram that captures the search and selection process.)

We then extracted data from all the remaining articles into a Google document (see Table 1 for the articles that met eligibility and the data we extracted from each). There was 1 case study, 1 mixed-methods study (both qualitative and quantitative measures), 12 qualitative studies and 37 quantitative studies. Of the quantitative studies, the majority were cross-sectional. There were nine studies reporting on mortality, all of which were cohort studies, some of which followed patients for years and some which used national or regional health records to determine mortality rates. The number of participants in the studies ranged from 1 to 72,021,918. The percentage of female participants ranged from 0% to 100%. For studies that provided a mean age of participants (n = 42), the average age ranged from 26.7 to 51.9 years. The most represented countries were India (n = 19), Ethiopia (n = 9) and China (n = 6), which made up two-thirds of the studies. Additional countries represented in the studies were Indonesia (n = 5), Nigeria (n = 3), Uganda (n = 3), Brazil (n = 2), Egypt (n = 2), Ghana (n = 1) and South Africa (n = 1). The most studied mental health condition was schizophrenia. The most studied traumatic event was interpersonal violence (76.5%–84.3%)^1^ including intimate partner violence and sexual and physical victimization types. Of the types of interpersonal violence, more than one-fifth were about physical restraint, which ranged from shackling people by their ankles to a tree to chemical restraint in a hospital (23.3%–34.9%)^1^. There were no studies on man-made or natural disasters.

**Table 1.** Articles and their characteristics included in the scoping review

First author – year of publication	Article title	Country	Region	Study design	Population description	# of participants	% Female	Mean age (years)	Race/ethnicity of participants	Type of trauma(s) assessed	How traumas were assessed	% of participants with SMHC who experienced traumatic event	Mental health diagnosis	How mental health diagnosis was assessed
Afe 2016 (Afe et al., 2016)	Intimate partner violence, psychopathology and the women with schizophrenia in an outpatient clinic South–South, Nigeria	Nigeria	Cross River State, South–South	Quantitative: cross-sectional	Female outpatients with SCZ	77	100%	38.3	Efik/Ibibio (49.0%), Ibos (34.0%), Yoruba (16.0%) and Hausas (1.0%)	IPV	WHO Violence Against Women Instrument	75%	SCZ	SCID
Aluh 2022 (Aluh et al., 2022)	Experiences and perceptions of coercive practices in mental health care among service users in Nigeria: a qualitative study	Nigeria	Not reported	Qualitative	Mental health service users at two major psychiatric hospitals	30	36.7%	34.67	Not reported	Coercive practices in hospitals, for example, use of ropes, chains and handcuffs for mechanical restraint, being whipped	Focus group discussions	40% chaining and 16.6% whipping	SCZ, SZA, BD, depression and other mental and behavioral disorders due to psychoactive substance	Not reported
Ametaj 2021 (Ametaj et al., 2021)	Traumatic Events and Posttraumatic Stress Disorder in Individuals with Severe Mental Illness in a Non-Western Setting: Data from Rural Ethiopia	Ethiopia	Sodo District, Gurage Zone	Qualitative	Patients with SMI, their caregivers, healthcare providers, and community and religious leaders in a rural community	48 in full sample; 13 with SMI	46.15% of people with SMI	35.15 years for people with SMI	Gurage (100%)	Multiple, including chaining, animal attacks, rape and physical assault	Semi-structured interview	N/A	SMI: SCZ, BD and depression with psychotic features	OPCRIT conducted by psychiatric nurses
An 2016 (An et al., 2016)	Physical restraint for psychiatric patients and its associations with clinical characteristics and the National Mental Health Law in China	China	Beijing	Quantitative: cross-sectional	Patients with psychiatric conditions	1,364	63.9%	36.2	Not reported	Physical restraint, for example, use of belts to fix a patient to a bed	Chart review and confirmed during interviews	35.2% of people with SCZ and 42.1% with mood disorders	SCZ, mood disorders and other	Confirmed in a clinical interview
Asher 2017 (Asher et al., 2017)	“I cry every day and night, I have my son tied in chains”: physical restraint of people with schizophrenia in community settings in Ethiopia	Ethiopia	Sodo and Butajira districts, Gurage Zone	Qualitative	People with SCZ, their caregivers, community leaders, and primary and community health workers in rural community	50 in full sample; 4 with SCZ	52.0% of full sample; 25% of people with SCZ	Not reported	N/A	Physical violence and restraint in community settings, for example, beating, use of handcuffs, chains and iron bars	Focus group discussions and in-depth interviews	N/A	SCZ	OPCRIT conducted by a psychiatric nurse
Bagewadi 2016 (Bagewadi et al., 2016)	Standardized Mortality Ratio in Patients with schizophrenia – Findings from Thirthahalli: A Rural South Indian Community	India	Thirthahalli, Karnataka	Quantitative: cohort	Patients with SCZ who live in a community setting who are alive and those who died	943 in full sample;12 deceased	33.3% of the deceased patients	45.1 for the deceased patients	Not reported	Accidents leading to death	Chart review and/or verbal report from family members	0.11% of the full cohort; 8.3% of the deceased patients	SCZ	Clinical diagnosis by a psychiatrist, followed by the MINI
Belete 2017 (Belete, 2017a)	Leveling and abuse among patients with bipolar disorder at psychiatric outpatient departments in Ethiopia	Ethiopia	Addis Ababa	Quantitative: cross-sectional	Outpatients with BD	411	57.4%	34.35	Amhara (34.55%), Oromo (32.85%), Gurage (20%), Tigray (5.8%) and others (6.8%)	Verbal or physical abuse	Chart review and by asking the patients and/or from family members	37.7%	BD	Clinical diagnosis by a psychiatrist or mental health professional specialist
Belete 2017 (Belete, 2017b)	Use of physical restraints among patients with bipolar disorder in Ethiopian Mental Specialized Hospital, outpatient department: cross-sectional study	Ethiopia	Addis Ababa	Quantitative: cross-sectional	Outpatients with BD	400	57.2%	32	Amhara (34.7%), Oromo (33.0%), Gurage (19.7%), Tigray (5.8%) and others (6.8%)	Physical restraint	Chart review and/or verbal report from family members	65%	BD	Clinical diagnosis by a psychiatrist or mental health professional specialist
Bhattacharya 2022 (Bhattacharya, 2022)	“The Day I Die Is the Day I Will Find My Peace”: Narratives of Family, Marriage, and Violence Among Women Living with Serious Mental Illness in India	India	Not reported	Qualitative	Current or former female inpatients with SMI living in an urban setting	11	100%	Mid‑30s to early 60s, no mean age provided	Not reported	Domestic and sexual violence	In-depth interviews	N/A	SMI: SCZ, SZA or BD	Not reported
Chandra 2003 (Chandra et al., 2003)	A Cry from the Darkness: Women with Severe Mental Illness in India Reveal Their Experiences with Sexual Coercion	India	Bangalore	Mixed-methods (qualitative and structured interviews)	Female inpatients with SMI	50	100%	30	Not reported	Sexual coercion, that is, forced sexual intercourse, unwanted sexual play and sexual experiences involving threats or use of physical force	Sexual Experiences Survey (SES) and semi-structured interview	34% coercive experiences after onset on mental illness	SMI: recurrent depressive disorder, SCZ spectrum disorder, BD and other disorders	Clinical diagnosis by a psychiatrist
Chandra 2003 (Chandra et al., 2003)	Sexual coercion and abuse among women with a severe mental illness in India: An exploratory investigation	India	Bangalore	Quantitative: cross-sectional	Female inpatients with SMI	146	100%	31.6	Not reported	Sexual coercion, that is, forced sexual intercourse, unwanted sexual play, and sexual experiences involving threats or use of physical force	Sexual Experiences Survey (SES)	16% of adult sexual coercion and 7% of both adult sexual coercion and child sexual abuse	SCZ-spectrum disorders, BD and depression	Clinical diagnosis by a psychiatrist
de Oliveira 2013 (de Oliveira et al., 2013)	Physical violence against patients with mental disorders in Brazil: sex differences in a cross-sectional study	Brazil	National	Quantitative: cross-sectional	Patients with SMI using public mental health services	2,475	51.6%	44.9 women and 51.9 men	Not reported	Physical violence	Semi-structured interview	12.0% of women and 16.0% of men experienced physical violence within health institutions by other patients, employees or health professionals. (Other physical violence reported could not be split out from lifetime exposure.)	SCZ and other psychosis, BD, depressive disorder and others	Chart review
El Missiry 2019 (El Missiry et al., 2019)	Rates and profile of victimization in a sample of Egyptian patients with major mental illness	Egypt	Cairo	Quantitative: cross-sectional	Inpatients and outpatients with major mental illness	300	52.0% of full sample	34.3 for victimized sample	Not reported	Covert/relational victimization or physical victimization, that is, threatened or subjected to corporeal damage	Victimization Questionnaire (VQ)	43.3%	Major mental illness: SCZ, BD and major depression	SCID
Fekadu 2015 (Fekadu et al., 2015)	Excess mortality in severe mental illness: 10-year population-based cohort study in rural Ethiopia	Ethiopia	Butajira District, Gurage Zone	Quantitative: cohort	People with SMI and those without in a rural community	919 in full cohort; 121 patients who died	37.8%	15–49 at baseline, no mean age provided	Not reported	Accidents leading to death and homicides	Verbal autopsy	9.10%	SMI: SCZ, BD and severe depression	SCAN conducted by a psychiatrist or mental health professional
Fekry 2012 (Fekry, 2011)	Clinical and sociodemographic profile of victimized versus nonvictimized Egyptian patients with bipolar mood disorder	Egypt	Cairo	Quantitative: cross-sectional	Inpatients and outpatients with BD	100	36.0% of full sample; 41.7% of victimized group	31	Not reported	Covert/relational victimization or physical victimization, that is, threatened or subjected to corporeal damage	Victimization Questionnaire (VQ)	48%	BD	SCID
Gilmoor 2020 (Gilmoor et al., 2020)	“If somebody could just understand what I am going through, it would make all the difference”: Conceptualizations of trauma in homeless populations experiencing severe mental illness	India	Kanchipuram and Chennai	Qualitative	People with SMI who were previously homeless or at risk of homelessness	26	76.9%	47	Not reported	Multiple, including abuse, man-made accidents, natural disasters, violence, illness and death	In-depth interviews and questions based on previous culturally relevant free listing exercise	N/A	SMI: SCZ, BD, psychosis NOS, substance induced delirium and major depressive disorder	Self-report
Gowda 2018 (Gowda et al., 2018)	Restraint prevalence and perceived coercion among psychiatric inpatients from South India: A prospective study	India	Bangalore	Quantitative: cohort	Inpatients with SCZ or mood disorders	200	45%	33.5	Not reported	Restraint measures: physical restraints, chemical restraints, seclusion/isolation and involuntary medication	Interviews with participants and health records	66.5% experienced 1+ restraint measures; 20% physical restraint, 58% chemical restraint, 18% seclusion and 32% involuntary medication	SCZ or other psychotic disorders and mood disorders	MINI
Hu 2022 (Hu et al., 2022)	A Retrospective Analysis of Death Among Chinese Han Patients with schizophrenia from Shandong	China	Shandong	Quantitative: cohort	Patients with SCZ who are alive and those who died	72,102 in full sample; 11,766 deceased	48.21%	47.21	Han (100%)	Accidents leading to death and homicides	Death certificates or forensic specialist	6.97%	SCZ	Clinical diagnosis by a psychiatrist
Jakhar 2015 (Jakhar et al., 2015)	A cross sectional study of prevalence and correlates of current and past risks in schizophrenia	India	New Delhi	Quantitative: cross-sectional	Patients with SCZ from the psychiatric ward of large mental hospitals	270	35.2%	34.0	Not reported	“Risk from others”	Ram Manohar Lohia Risk Assessment Interview	11%	SCZ	Referral by treating a clinician followed by Diagnostic Interview for Genetic Studies – Hindi version (DIGS)
Lundberg 2012 (Lundberg et al., 2012b)	Sexual Risk Behaviors and Sexual Abuse in Persons with Severe Mental Illness in Uganda: A Qualitative Study	Uganda	Kampala	Qualitative	Inpatients cleared for discharge and outpatients	20	65%	18–49, no mean age provided	Not reported	Sexual abuse	Semi-structured interview	N/A	SMI: SCZ, BD or depression	Chart review
Lundberg 2015 (Lundberg et al., 2015)	Sexual Risk Behavior, Sexual Violence, and HIV in Persons with Severe Mental Illness in Uganda: Hospital-Based Cross-Sectional Study and National Comparison Data	Uganda	Kampala	Quantitative: cross-sectional	Former inpatients with SMI	602	57.0% (note: only women assessed for sexual violence)	18–49, no mean age provided	Not reported	Sexual violence	WHO Violence Against Women Instrument	24.2% of women experienced sexual violence by a partner; 10.5% of women experienced sexual violence by a non-partner	SMI: BD, nonaffective psychosis and major depression	Chart review
Manjunatha 2019 (Manjunatha et al., 2019)	Mortality in schizophrenia: A study of verbal autopsy from cohorts of two rural communities of South India	India	Thirthahalli and Turuvekere, Karnataka	Quantitative: cohort	People with SCZ from rural communities who died	53	47.1%	50.5	Not reported	Death due to road traffic accidents	Verbal autopsy	5.6%	SCZ	Clinical diagnosis by a psychiatrist, followed by the MINI
Melo 2022 (Melo et al., 2022)	All-cause and cause-specific mortality among people with severe mental illness in Brazil’s public health system, 2000–2015: a retrospective study	Brazil	National	Quantitative: cohort	Inpatients with SMI and those without in the public health system	72,021,918 in full sample; 749,720 with SMI	56.9% of the full sample and 50.5% of the people with SMI	41.1 for full sample	Not reported	Injuries including interpersonal violence and unintentional causes such as fires, drowning, foreign body, road injuries and falls	Chart review	19.9% of deaths in the population with SMI	SMI: SCZ, SZA, BD or depressive disorder	Chart review
Minas 2008 (Minas and Diatri, 2008)	Pasung: Physical restraint and confinement of the mentally ill in the community	Indonesia	Samosir	Quantitative: cross-sectional	People with SMI in the community	15	46.7%	25–56 years, no mean age provided	Not reported	Physical restraint and confinement, that is, by wooden stocks, confined in a small room or hut, tied with rope or chained	Physical identification (case finding)	100%	SCZ and other	Self-report and reports by family or community members
Mojtabai 2001 (Mojtabai et al., 2001)	Mortality and long-term course in schizophrenia with a poor 2-year course: A study in a developing country	India	Urban and rural Chandigarh	Quantitative: cohort	Patients with SCZ in an urban and a rural setting	171; 24 participants who died	47% of full sample	26.7 at baseline	Not reported	Death due to traffic accidents	Verbal autopsy and/or medical records	0.58% of full sample; 4.2% of the deceased group	SCZ	Present State Examination (PSE)
Moodley 2023 (Moodley et al., 2023)	The missed pandemic: Intimate partner violence in female mental-health-care-users during the COVID‑19 pandemic	South Africa	KwaZulu-Natal	Quantitative: cross-sectional	Female outpatients with SMI	154	100%	42.7	Black (50.3%), White (17.2%), Indian (21.2%) and colored (11.3%)	Interpersonal violence	Women Abuse Screening Tool (WAST)	46.6%	SMI: SCZ spectrum disorder, BD, major depressive disorder, anxiety disorders and personality disorders	Diagnoses were confirmed clinically using DSM‑5 criteria by the researcher and chart reviews provided by the treating doctor
Mpango 2023 (Mpango et al., 2023)	Physical and sexual victimization of persons with severe mental illness seeking care in central and southwestern Uganda	Uganda	Kampala and Masaka	Quantitative: cross-sectional	Outpatients with SMI	1,201	54.5%	36	Not reported	Physical and sexual victimization	Adverse life events module of the European Para-suicide Interview Schedule (EPSIS I)	13.6% physical victimization and 8.6% sexual victimization in the last 12 months	SMI: SCZ, BD and recurrent major depressive disorder	Chart review followed by the MINI
Nair 2020 (Nair et al., 2020)	Prevalence and clinical correlates of intimate partner violence (IPV) in women with severe mental illness (SMI)	India	“Southern part of India”	Quantitative: cross-sectional	Female inpatients with SMI who cohabitate with a partner	100	100%	34.2	Not reported	IPV	Indian Family Violence and Control Scale (IFVCS)	20% in the last 12 months	Psychosis and BD	Chart review
Ng 2019 (Ng et al., 2019)	Trauma exposure, depression, suicidal ideation, and alcohol use in people with severe mental disorder in Ethiopia	Ethiopia	Sodo District, Gurage zone	Quantitative: cross-sectional	People with severe mental disorders in a rural community	300	43%	36	Not reported	Physical restraint, assault, being beaten, rape, and more	List of Threatening Experiences scale (LTE); 2 questions on restraint; 13 questions on locally established traumatic events, for example, rape, beaten and hit by car	26.76% serious illness/injury-self; 46.33% restrained; 35.67% locally relevant traumatic events	Primary psychosis, BD and depression with psychotic features	OPCRIT conducted by psychiatric nurses
Opekitan 2017 (Opekitan et al., 2017)	Socio-Demographic Characteristics, Partner Characteristics, Socioeconomic Variables, and Intimate Partner Violence in Women with schizophrenia in South–South Nigeria	Nigeria	South–South	Quantitative: cross-sectional	Female outpatients with SCZ	79	100%	38.3	Efik/Ibibio (51.0%), Ibos (33.0%), Yorubas (15.0%) and Hausas (1.0%)	IPV	WHO Violence Against Women Instrument	73.0%	SCZ	SCID
Paul 2018 (Paul, 2018)	Are we doing enough? Stigma, discrimination and human rights violations of people living with schizophrenia in India: Implications for social work practice	India	Mumbai	Qualitative	People with SCZ or SCZ-related disorders, family members and mental health professionals	40 in full sample; 20 with SCZ-related disorder	50% of people with SCZ	40.85 for people with SCZ	Not reported	Stigma, discrimination and human rights violations, including physical and emotional violence	In-depth interviews and focus group discussions	N/A	SCZ and/or a SCZ-related disorder	Not reported
Ponnudurai 2006 (Ponnudurai et al., 2006)	Assessment of mortality and marital status of schizophrenic patients over a period of 13 years	India	Tamil Nadu	Quantitative: cohort	Outpatients with SCZ who died	60	11.67%	27 for men and 26.7 for women at baseline	Not reported	Death due to accidents	Case notes at the hospital and verbal autopsies confirmed by police reports	1.67%	SCZ	Independently confirmed by two psychiatrists
Ran 2007 (Ran et al., 2007)	Mortality in people with schizophrenia in rural China: 10-year cohort study	China	Xinjin County, Chengdu	Quantitative: cohort	Patients with SCZ in a rural community who are alive and those who died	500; 98 participants who died	53.4% of full sample	44.7	Not reported	Death due to accidents	Death certificates and verbal autopsies	2.6% of full sample; 13.3% of the deceased group	SCZ	Present State Examination (PSE)
Rani 2023 (Rani et al., 2023)	A Qualitative Study to Understand the Nature of Abuse Experienced by Persons with Severe Mental Illness	India	Bengaluru	Qualitative	Outpatients with SMIs, their caregivers and experts from the community	51 in full sample; 14 with SMI	50% of people with SMI	18–60+, no mean age provided	Not reported	Physical abuse, psychological abuse, sexual abuse, social abuse and trauma in formal care	Key informant interviews and focus group discussions	N/A	SMI: BD, SCZ, SZA and recurrent depressive disorder	Not reported
Rani 2023 (Rani et al., 2023)	Profiles of Victimized Outpatients with Severe Mental Illness in India	India	Not reported	Quantitative: cross-sectional	Outpatients with SMI	150	56%	36.16	Not reported	Emotional abuse, severe combined abuse, physical abuse and harassment	Composite Abuse Scale (CAS)	100% experienced emotional abuse, 94% severe combined abuse, 92.7% physical abuse and 54% harassment in the past 12 months	SMI: BD, SCZ, SZA and recurrent depressive disorder	Chart review
Read 2009 (Read et al., 2009)	Local suffering and the global discourse of mental health and human rights: An ethnographic study of responses to mental illness in rural Ghana	Ghana	Kintampo	Qualitative and ethnographic methods	People with SMI, their families, healing practitioners and religious leaders within rural communities	67 in full sample; 25 with SMI	Not reported	Not reported	Not reported	Chaining, beating and withholding of food	Observation, semi-structured interviews and focus group discussions	N/A	SMI	Not reported
Reddy 2020 (Reddy et al., 2020)	Childhood abuse and intimate partner violence among women with mood disorders	India	Bangalore	Quantitative: cross-sectional	Women with SMI vs. healthy women with intimate partners	251 with SMI and 72 healthy women	100%	35.35 for people with SMI	Not reported	Childhood abuse and IPV	Composite Abuse Scale to assess the adulthood IPV	29.1% severe combined abuse; 37.5% emotional abuse; 47.8% physical abuse; 25.9% harassment in the population with SMI	SMI: unipolar depression and BD	Clinical diagnosis by a psychiatrist, followed by the MINI
Sadath 2014 (Sadath et al., 2014)	Human rights violation in mental health: A case report from India	India	“South India”	Case report	Man with SCZ	1	0%	41	Not reported	Solitary confinement in a single room/terrace with no ventilation, sanitary facilities, fed through window and personal care not given for >15 years	Physical identification (case finding)	100%	Paranoid SCZ	Not reported
Sam 2019 (Sam et al., 2019)	Stressful Life Events and Relapse in Bipolar Affective Disorder: A Cross-Sectional Study from a Tertiary Care Center of Southern India	India	Kolenchery, Kerala	Quantitative: cross-sectional	Inpatients with BD	128	43.0%	40.19	Not reported	Major personal illness or injury	Presumptive Stressful Life Events Scale (PSLES)	3.4%	BD: mania and depression	Not reported, beyond stating “ICD‑10 criteria”
Subramanian 2017 (Subramanian et al., 2017)	Role of stressful life events and kindling in bipolar disorder: Converging evidence from a mania-predominant illness course	India	“Southern India”	Quantitative: cross-sectional	Inpatients and outpatients with BD	149	52.3%	37.7	Not reported	Major personal illness or injury	Presumptive Stressful Life Events Scale (PSLES)	0.8%	BD	SCID
Suryani 2011 (Suryani et al., 2011)	Treating the untreated: Applying a community-based, culturally sensitive psychiatric intervention to confined and physically restrained mentally ill individuals in Bali, Indonesia	Indonesia	Karangasem regency, Bali	Quantitative: cross-sectional	People with SCZ-spectrum disorders in the community	23	13%	45.1	Not reported	Restraint with iron shackles, ropes, wooden stocks or wooden cages	Physical identification (case finding)	91.3% restraint with iron shackles, ropes and wooden stocks; 8.7% confined to wooden cages	SCZ-spectrum disorder	Clinical diagnosis by a psychiatrist
Teferra 2011 (Teferra et al., 2011)	Five-year mortality in a cohort of people with schizophrenia in Ethiopia	Ethiopia	Butajira District, Gurage zone	Quantitative: cohort	Patients with SCZ in a rural community who are alive and those who died	307; 38 participants who died	17.9% of the full sample; 10.5% of the deceased group	35 for the deceased group	Not reported	Death due to traffic accidents	Verbal autopsy	0.7% of full sample; 5.3% of the deceased group	SCZ	SCAN conducted by a psychiatrist or mental health professional
Tsigebrhan 2014 (Tsigebrhan et al., 2014)	Violence and violent victimization in people with severe mental illness in a rural low-income country setting: A comparative cross-sectional community study	Ethiopia	Butajira District, Gurage Zone	Quantitative: cross-sectional	People with SMI and those without in a rural community	401 in full sample; 201 with SMI and 200 without	38.8% in the full sample	40 for the full sample; 40.3 for people with SMI	Not reported	Violent victimization, for example, being kicked, dragged, chained or beaten; threatened or attacked with a weapon; forced to have sexual intercourse	Adapted version of the McArthur Violence Interview	17.4% in the past 12 months	SMI: SCZ, SZA and BD	SCAN conducted by a psychiatrist or mental health professional
Vijayalakshmi 2012 (Vijayalakshmi et al., 2012)	Gender-Related Differences in the Human Rights Needs of Patients with Mental Illness	India	Not reported	Quantitative: cross-sectional	Asymptomatic outpatients with SCZ or mood disorders	100	47.0%	34.68	Not reported	Torture, cruel, inhuman or degrading treatment or punishment; not allowed to leave home; sexual advances by family members	Needs assessment questionnaire based on Universal Declaration of Human Rights	61.0% sexual advances by family members and 35.0% not allowed to leave their home	Mood disorders or SCZ disorders	Not reported
Wang 2020 (Wang et al., 2020)	Frequency and correlates of violence against patients with schizophrenia living in rural China	China	Luoding County, Guangdong	Quantitative: cross-sectional	Outpatients with SCZ in a rural community	487	35.9%	42.36	Not reported	Violent victimization in the past 6 months, for example, sexual assault with violence, sexual harassment with physical contacts, verbal harassment with sexual content, nonsexual physical violence and verbal threat and abuse by family members or others	Asked participants	18.9% experienced 1+ event in the past 6 months	SCZ	Chart reviewed followed by a clinical interview
Windarwati 2021 (Windarwati et al., 2021)	A Journey of Hidden Outburst of Anger Shackling a Person with schizophrenia: The Indonesian Context	Indonesia	East Java	Qualitative	People with SCZ, family members, volunteers, prominent figures and nurses	23 in full sample; 5 with SCZ	0% of people with SCZ	> 40, no mean age provided	Not reported	Shackling by ankles	Records from regional health office and grounded-theory approach	100%	SCZ	Not reported
Yosep 2021 (Yosep et al., 2021)	How patients with schizophrenia “as a Victim” cope with violence in Indonesia: a qualitative study	Indonesia	West Java	Qualitative	Patients with SCZ from the psychiatric ward of large mental hospitals	40	35.0%	35.8	Not reported	Pushing, punching, kicking and restraining	Semi-structured interview	100%	SCZ	Confirmed diagnosis by a physician
Yosep 2021 (Yosep et al., 2021)	Experiences of Violence Among Individuals With schizophrenia in Indonesia a Phenomenological Study	Indonesia	West Java	Qualitative	Patients with SCZ recruited from referral hospitals	40	37.5%	35.6	Not reported	Victimization by nurses and family members, including physical and verbal violence	Focus group discussions	N/A	SCZ	Not reported
Zerihun 2021 (Zerihun et al., 2021)	Intimate partner violence among reproductive-age women with chronic mental illness attending a psychiatry outpatient department: cross-sectional facility-based study, Addis Ababa, Ethiopia	Ethiopia	Addis Ababa	Quantitative: cross-sectional	Female outpatients of reproductive age	422	100%	32.1	Not reported	IPV	WHO Violence Against Women Instrument	44.1% in the past 12 months	SCZ, BD, and severe major depression	Participants recruited from outpatient departments, but diagnosis process not stated
Zhang 2018 (Zhang et al., 2018)	Long-Term Outcomes of Unlocking Chinese Patients with Severe Mental Illness	China	Hebei	Quantitative: cohort	Patients with SMI	107	16.82% at baseline	35.9 at baseline	Not reported	Physical restraint, for example, use of iron chains, iron cage, rope or a separate room or shed	Not reported	100% at baseline; 19.6% at Time 2 (year 2012); 17.8% at Time 3 (year 2016)	SMI: SCZ, paranoid psychosis and BD	Not reported, beyond stating “ICD‑10 criteria”
Zhu 2014 (Zhu et al., 2014)	Frequency of Physical Restraint and Its Associations with Demographic and Clinical Characteristics in a Chinese Psychiatric Institution	China	Hunan	Quantitative: cross-sectional	Inpatients with psychiatric conditions	160; 85 with SCZ and BP	50.6% of full sample	30.0	Not reported	Physical restraint	Medical records and confirmed by interview	51.3%	Of the full sample: SCZ, mood disorders and others	Chart review and confirmed by clinical interview

Acronyms: BP = bipolar disorder; DSM = Diagnostic and Statistical Manual of Mental Disorders; ICD = International Classification of Diseases; IPV = intimate partner violence; MINI = Mini International Neuropsychiatric Interview; N/A = not applicable; OPCRIT = Operational Criteria Checklist for Psychotic Illness; PSE = Present State Examination; SCID = Structured Clinical Interview for DSM-IV; SCZ = schizophrenia; SMI = severe mental illness or serious mental illness; SZA = schizoaffective disorder; WHO = World Health Organization.

Note: the terminology in the table reflects the language the researchers used in their articles, such as SMI.

## Discussion

This scoping review aimed to synthesize the existing literature on trauma exposure experienced by adults with preexisting SMHCs in LMICs. By including multiple types of studies, we captured a breadth of trauma types and SMHCs that inform the different ways we conceptualize trauma exposure across mental health diagnoses in LMIC settings. The range of sample sizes, sexes represented, age of participants and variation in diagnoses also illustrates the diversity of studies in the field, which may make the findings more reflective of real-world experiences of patients with SMHCs.

Of the 51 included articles, India, Ethiopia and China made up more than 66% of the studies; thus, even though there was a broad range of types of studies and demographics, our understanding of the field derives from a small subset of countries. For example, eight of the nine mortality studies were from India, Ethiopia and China, providing data on traumas that led to death in these populations. Furthermore, with only 10 countries represented in total, there were no data from the more than 120 other LMICs. Though the additional articles outside of India, Ethiopia and China included studies from Southeast Asia, South America and Africa, these regions and the countries within them have diverse populations, geographies and cultures. As such, the studies included may not reflect the full scope of types and frequencies of traumas that people with SMHCs face within and across such heterogeneous LMICs.

Interpersonal violence, which accounted for the majority of studies, emerged as a significant category of traumatic events. Such studies support the notion that people with SMHCs are at additional risk for victimization given their mental health conditions. For example, having an SMHC is associated with social and physical isolation and potential cognitive impairments and/or symptoms (i.e., positive and negative symptoms), which increase the likelihood of being targeted by a perpetrator (de Vries et al., 2019). Alternatively, in many studies family members said they had tied up their loved ones in order to protect them from harm in the community and had no other options given the lack of treatment options and accessible and affordable health care (Asher et al., 2017).

One type of interpersonal violence, “chaining” and “restraining,” represented a significant percentage of events. However, the type of restraint differed widely. While some participants were kept in wooden cages in their community for years, some were in solitary isolation as inpatients for a short period. In both high-income countries (the United States) and in LMICs (Ethiopia and China), people with SMHCs have described restraint as traumatizing (Frueh et al., 2005; Zhu et al., 2014; Ametaj et al., 2021). In Uganda, the United Nations Office of the High Commissioner for Human Rights has described the use of physical, mechanical and chemical restraints and isolation/seclusion in psychiatric facilities in conflict with the Convention on the Rights of Persons with Disabilities’ Article 15 (“Freedom from torture and cruel, inhuman or degrading treatment or punishment” for all people) (OHCHR, 2016).

Even in nonpsychiatric contexts, there is evidence that physical restraint can lead to physical and psychological harm, including PTSD (Franks et al., 2021). The concept of restraint can be controversial, however, because it can be deemed necessary by hospital staff for a patient’s safety depending on an institution’s clinical guidelines. Restraint is also not captured by measures that are often used to assess trauma exposure such as the LEC and the CIDI. Such standardized measures can sometimes fail to capture a wider range of exposures as well as the subjective nature of what is considered traumatic. This may have affected how participants reported traumatic events in these countries.

This is particularly relevant in non-Western settings. Some researchers maintain that the acknowledgment of traumatic events and subsequent traumatic stress is not simply a response to objective experiences, but is shaped by the culture in which one exists and is understood through what is considered traumatic in their setting (Chentsova-Dutton and Maercker, 2019). The value of including qualitative research is that it can augment quantitative reports by offering insights into personal trauma experiences and contextual factors in these settings.

Surprisingly, there were no studies about man-made or natural disasters, such as war, fires and landslides. There is literature about vulnerable populations being left behind in dangerous situations because they are incapable of fleeing, such as the elderly in conflict zones (Murthy and Lakshminarayana, 2006) or during environmental crises like Hurricane Katrina (Dosa et al., 2010). However, none of these trauma types were elicited in this search. It is possible that this is because it is harder to conduct research during events like wars (Cohen and Arieli, 2011), and this absence partially reflects a survival bias. That is, people with SMHCs who died during disasters may not be represented in studies. Despite this, there were mortality studies in the scoping review that captured types of traumas that led to death, somewhat counterbalancing this potential survival bias. Such studies were conducted in more controlled environments in which populations could be followed for years or in countries where there were strong national or regional electronic medical records where accurate patient outcome data could be pulled more easily.

## Limitations

There are some limitations that should be noted here. Although we tried to capture an extensive range of trauma types, we know that there are traumas that are culturally relevant in the countries we looked at that might not meet DSM-5 or ICD-11 criteria. While we tried to account for this by including “idioms of distress” and terminology about human rights violations and chaining/restraint, it is likely that there were trauma types that have not been studied by researchers in these contexts or that used different terms from ours that we did not retrieve. In addition, if an article did not include the name of the country in its title or abstract or a grouping term for the countries that met our criteria (i.e., “LMIC” or “East Africa”), we would likely have missed it. We may have missed articles that were not indexed in the American or European databases used here, as some articles from LMICs do not get included in these databases. However, we included Africa-Wide Information/NiPad in order to capture some of the articles that might have been otherwise missed. We limited inclusion to published studies in the interest of completing this scoping review in a timely manner. We acknowledge that excluding gray literature may potentially introduce publication bias. Inclusion of gray literature to address this research question should be considered in a future systematic review. Lastly, we did not include articles with potential mediators between SMHCs and trauma exposure, such as substance use or economic disadvantage. Studies with mediators would be worthwhile to look at in the future.

## Conclusion

Future research on trauma types that people with SMHCs are exposed to in LMICs should expand beyond 10 countries to more than 120 additional countries that qualify as LMICs. Research on man-made and natural disasters in this population is warranted. Additional qualitative research may help identify locally relevant trauma types as well as understand people’s lived experiences.

## Supporting information

Stevenson et al. supplementary material Stevenson et al. supplementary material

## Data Availability

No new data were created as part of this manuscript. The authors confirm that the data supporting the findings of this study are available within the article.

## Footnotes

1 The ranges here (76.5%–84.3% and 23.3%–34.9%) reflect that some studies collapsed many trauma types into one field. Thus, types of interpersonal violence could not always be teased out from non-interpersonal violence, such as sexual violence vs. road traffic accidents and physical restraint could not always be teased out from non-physical restraint.

## Supplementary material

The supplementary material for this article can be found as a Word document, which is attached to this article.

## References

[r1] Afe, T. O., Emedoh, T. C., Ogunsemi, O., & Adegbohun, A. A. (2016). Intimate partner violence, psychopathology and the women with schizophrenia in an outpatient clinic south-south, Nigeria. BMC Psychiatry, 16(1), 197. 10.1186/s12888-016-0898-2. 27287452 PMC4902947

[r2] Alem, A. (2000). Human rights and psychiatric carein Africa with particular referenceto the Ethiopian situation. Acta Psychiatrica Scandinavica, 101(399), 93–96. 10.1111/j.0902-4441.2000.007s020[dash]21.x. 10794037

[r3] Aluh, D. O., Ayilara, O., Onu, J. U., Pedrosa, B., Santos-Dias, M., Cardoso, G., Caldas-de-Almeida, J. M., & Grigaite, U. (2022). Experiences and perceptions of coercive practices in mental health care among service users in Nigeria: A qualitative study. International Journal of Mental Health Systems, 16(1). 10.1186/s13033-022-00565-4. PMC9694572 36424651

[r4] American Psychiatric Association. (2013). Diagnostic and Statistical Manual of Mental Disorders: DSM-5™, 5th ed. American Psychiatric Publishing, Inc. 10.1176/appi.books.9780890425596.

[r5] Ametaj, A. A., Hook, K., Cheng, Y. H., Serba, E. G., Koenen, K. C., Fekadu, A., & Ng, L. C. (2021). Traumatic events and posttraumatic stress disorder in individuals with severe mental illness in a non-Western setting: Data from rural Ethiopia. Psychological Trauma-Theory Research Practice and Policy, 13(6), 684–693. 10.1037/tra0001006. 33539160 PMC8333189

[r6] An, F. R., Sha, S., Zhang, Q. E., Wu, P. P., & Jin, X. (2016). Physical restraint for psychiatric patients and its associations with clinical characteristics and the National Mental Health Law in China. Psychiatry Research, 241 (An F.-R.; Sha S.; Zhang Q.-E.; Wu P.-P.; Jin X.) National Clinical Research Center for Mental Disorders & Beijing Anding Hospital, Capital Medical University, China(Ungvari G.S.) The University of Notre Dame Australia/Marian Centre, Perth, Australia(Ungv), 154–158. 10.1016/j.psychres.2016.04.101. 27179180

[r7] Asher, L., Fekadu, A., Teferra, S., De Silva, M., Pathare, S., & Hanlon, C. (2017). “I cry every day and night, I have my son tied in chains”: Physical restraint of people with schizophrenia in community settings in Ethiopia. Globalization and Health, 13. 10.1186/s12992-017-0273-1. PMC5504711 28693614

[r8] Bagewadi, V. I., Kumar, C. N., Thirthalli, J., Rao, G. N., Suresha, K. K., & Gangadhar, B. N. (2016). Standardized mortality ratio in patients with schizophrenia – Findings from Thirthahalli: A rural south Indian community. Indian Journal of Psychological Medicine, 38(3), 202–206. 10.4103/0253-7176.183083. 27335514 PMC4904755

[r9] Belete, H. (2017a). Leveling and abuse among patients with bipolar disorder at psychiatric outpatient departments in Ethiopia. Annals of General Psychiatry, 16, 29. 10.1186/s12991-017-0152-4. 28702070 PMC5505138

[r10] Belete, H. (2017b). Use of physical restraints among patients with bipolar disorder in Ethiopian mental specialized hospital, outpatient department: Cross-sectional study. International Journal of Bipolar Disorders, 5. 10.1186/s40345-017-0084-6. PMC5362568 28332124

[r11] Bhattacharya, A. (2022). “The day I die is the day I will find my peace”: Narratives of family, marriage, and violence among women living with serious mental illness in India. Violence Against Women, 28(3–4), 966–990. 10.1177/10778012211012089. 34120536

[r12] Chandra, P. S., Carey, M. P., Carey, K. B., Shalinianant, A., & Thomas, T. (2003). Sexual coercion and abuse among women with a severe mental illness in India: An exploratory investigation. Comprehensive Psychiatry, 44(3), 205–212. 10.1016/S0010-440X(03)00004-X. 12764708 PMC2424186

[r13] Chandra, P. S., Deepthivarma, S., Carey, M. P., Carey, K. B., & Shalinianant, M. P. (2003). A cry from the darkness: Women with severe mental illness in India reveal their experiences with sexual coercion. Psychiatry-Interpersonal and Biological Processes, 66(4), 323–334. 10.1521/psyc.66.4.323.25446. PMC2430935 14964694

[r14] Chentsova-Dutton, Y., & Maercker, A. (2019). Cultural scripts of traumatic stress: Outline, illustrations, and research opportunities [Hypothesis and theory]. Frontiers in Psychology, 10. 10.3389/fpsyg.2019.02528. PMC6872530 31803094

[r15] Cohen, N., & Arieli, T. (2011). Field research in conflict environments: Methodological challenges and snowball sampling. Journal of Peace Research, 48(4), 423–435. 10.1177/0022343311405698.

[r80] Cumpston, M., Lasserson, T., Flemyng, Ella., & Page, M.J., (2022). Cochrane Handbook for Systematic Reviews of Interventions version 6.3: Chapter III: Reporting the review. https://training.cochrane.org/handbook/current/chapter-iii. Accessed March 19, 2023.

[r16] de Oliveira, H. N., Machado, C. J., & Guimaraes, M. D. C. (2013). Physical violence against patients with mental disorders in Brazil: Sex differences in a cross-sectional study. Revista de Psiquiatria Clinica, 40(5), 172–176. 10.1590/S0101-60832013000500002.

[r17] de Vries, B., van Busschbach, J. T., van der Stouwe, E. C. D., Aleman, A., van Dijk, J. J. M., Lysaker, P. H., Arends, J., Nijman, S. A., & Pijnenborg, G. H. M. (2019). Prevalence rate and risk factors of victimization in adult patients with a psychotic disorder: A systematic review and Meta-analysis. Schizophrenia Bulletin, 45(1), 114–126. 10.1093/schbul/sby020. 29547958 PMC6293237

[r18] Dosa, D., Feng, Z., Hyer, K., Brown, L. M., Thomas, K., & Mor, V. (2010). Effects of hurricane Katrina on nursing facility resident mortality, hospitalization, and functional decline. Disaster Medicine and Public Health Preparedness, 4 Suppl 1(0 1), S28–32. 10.1001/dmp.2010.11. 23105032 PMC3773950

[r19] Dundar, Y., & Fleeman, N. (2017). Developing my search strategy. In A. Boland, R. Dickson, & G. Cherry (Eds.), Doing a Systematic Review (pp. 61–78). Sage.

[r20] El Missiry, A., El Meguid, M. A., Abourayah, A., El Missiry, M., Hossam, M., Elkholy, H., & Khalil, A. H. (2019). Rates and profile of victimization in a sample of Egyptian patients with major mental illness. International Journal of Social Psychiatry, 65(3), 183–193. 10.1177/0020764019831315. 30848686

[r21] Fekadu, A., Medhin, G., Kebede, D., Alem, A., Cleare, A. J., Prince, M., Hanlon, C., & Shibre, T. (2015). Excess mortality in severe mental illness: 10-year population-based cohort study in rural Ethiopia. The British Journal of Psychiatry, 206(4), 289–296. 10.1192/bjp.bp.114.149112. 25657358

[r22] Fekry, M. (2011). Clinical and psychodemographic profile of victimized versus nonvictimized Egyptian patients with bipolar mood disorder. Middle East Current Psychiatry, 19, 131–141.

[r23] Franks, Z. M., Alcock, J. A., Lam, T., Haines, K. J., Arora, N., & Ramanan, M. (2021). Physical restraints and post-traumatic stress disorder in survivors of critical illness. A systematic review and Meta-analysis. Annals of the American Thoracic Society, 18(4), 689–697. 10.1513/AnnalsATS.202006-738OC. 33075240

[r24] Frueh, C., Knapp, R. G., Cusack, K. J., Grubaugh, A. L., Sauvageot, J. A., Cousins, V. C., Yim, E., Robins, C. S., Monnier, J., & Hiers, T. G. (2005). Special section on seclusion and restraint: Patients’ reports of traumatic or harmful experiences within the psychiatric setting. Psychiatric Services, 56(9), 1123–1133. 10.1176/appi.ps.56.9.1123. 16148328

[r25] Gilmoor, A. R., Vallath, S., Regeer, B., & Bunders, J. F. G. (2020). “If somebody could just understand what I am going through, it would make all the difference”: Conceptualizations of trauma in homeless populations experiencing severe mental illness. Transcultural Psychiatry, 57(3), 455–467. 10.1177/1363461520909613. 32148189 PMC7263042

[r26] Gowda, G. S., Lepping, P., Noorthoorn, E. O., Ali, S. F., Kumar, C. N., Raveesh, B. N., & Math, S. B. (2018). Restraint prevalence and perceived coercion among psychiatric inpatients from South India: A prospective study. Asian Journal of Psychiatry, 36, 10–16. 10.1016/j.ajp.2018.05.024. 29857265

[r27] Grubaugh, A. L., Zinzow, H. M., Paul, L., Egede, L. E., & Frueh, B. C. (2011). Trauma exposure and posttraumatic stress disorder in adults with severe mental illness: A critical review. Clinical Psychology Review, 31(6), 883–899. 10.1016/j.cpr.2011.04.003. 21596012 PMC3148295

[r28] Guan, L., Liu, J., Wu, X. M., Chen, D., Wang, X., Ma, N., Wang, Y., Good, B., Ma, H., Yu, X., & Good, M. J. (2015). Unlocking patients with mental disorders who were in restraints at home: A national follow-up study of china’s new public mental health initiatives [Article]. PLoS One, 10(4). 10.1371/journal.pone.0121425. PMC4388503 25848800

[r29] Hidayat, M. T., Oster, C., Muir-Cochrane, E., & Lawn, S. (2023). Indonesia free from pasung: A policy analysis. International Journal of Mental Health Systems, 17(1), 12. 10.1186/s13033-023-00579-6. 37138360 PMC10155453

[r30] Hu, L. L., Wang, Q., Wang, Y. H., Gu, L. X., & Yu, T. G. (2022). A retrospective analysis of death among Chinese Han patients with schizophrenia from Shandong. Risk Management and Healthcare Policy, 15, 403–414. 10.2147/RMHP.S351523. 35300275 PMC8923028

[r31] Jakhar, K., Bhatia, T., Saha, R., & Deshpande, S. N. (2015). A cross sectional study of prevalence and correlates of current and past risks in schizophrenia. Asian Journal of Psychiatry, 14, 36–41. 10.1016/j.ajp.2015.01.005. 25703039 PMC4450129

[r32] Kessler, R. C., & Üstün, T. B. (2004). The World Mental Health (WMH) survey initiative version of the World Health Organization (WHO) Composite International Diagnostic Interview (CIDI). International Journal of Methods in Psychiatric Research, 13(2), 93–121. 10.1002/mpr.168. 15297906 PMC6878592

[r33] Lundberg, P., Johansson, E., Okello, E., Allebeck, P., & Thorson, A. (2012a). Sexual risk behaviours and sexual abuse in persons with severe mental illness in Uganda: A qualitative study. PLoS One, 7(1), e29748. 10.1371/journal.pone.0029748. 22253770 PMC3253795

[r34] Lundberg, P., Nakasujja, N., Musisi, S., Thorson, A. E., Cantor-Graae, E., & Allebeck, P. (2015). Sexual risk behavior, sexual violence, and HIV in persons with severe mental illness in Uganda: Hospital-based cross-sectional study and National Comparison Data. American Journal of Public Health, 105(6), 1142–1148. 10.2105/AJPH.2014.302479. 25880958 PMC4431108

[r35] Maniglio, R. (2009). Severe mental illness and criminal victimization: A systematic review. Acta Psychiatrica Scandinavica, 119(3), 180–191. 10.1111/j.1600-0447.2008.01300.x. 19016668

[r36] Manjunatha, N., Kumar, C. N., Thirthalli, J., Suresha, K. K., Harisha, D. M., & Arunachala, U. (2019). Mortality in schizophrenia: A study of verbal autopsy from cohorts of two rural communities of South India. Indian Journal of Psychiatry, 61(3), 238–243. 10.4103/psychiatry.IndianJPsychiatry_135_19. 31142900 PMC6532470

[r37] Mauritz, M. W., Goossens, P. J. J., Draijer, N., & van Achterberg, T. (2013). Prevalence of interpersonal trauma exposure and trauma-related disorders in severe mental illness. European Journal of Psychotraumatology, 4(1), 19985. 10.3402/ejpt.v4i0.19985. PMC3621904 23577228

[r38] Melo, A. P. S., Dippenaar, I. N., Johnson, S. C., Weaver, N. D., Acurcio, F. D. A., Malta, D. C., Ribeiro, A. L. P., Guerra, A. A., Jr., Wool, E. E., Naghavi, M., & Cherchiglia, M. L. (2022). All-cause and cause-specific mortality among people with severe mental illness in Brazil’s public health system, 2000-15: A retrospective study. Lancet Psychiatry, 9(10), 771–781. 10.1016/S2215-0366(22)00237-1. 35964638 PMC9477749

[r39] Mercy, J., Hillis, S., Butchart, A., Bellis, M., Ward, C., Fang, X., & Rosenberg, M. (2017). Interpersonal Violence: Global Impact and Paths to Prevention (3rd ed.). The International Bank for Reconstruction and Development/The World Bank. 10.1596/978-1-4648-0522-6_ch5. 30212109

[r40] Minas, H., & Diatri, H. (2008). Pasung: Physical restraint and confinement of the mentally ill in the community [Article]. International Journal of Mental Health Systems, 2. 10.1186/1752-4458-2-8. PMC2442049 18554420

[r41] Mojtabai, R., Varma, V. K., Malhotra, S., Mattoo, S. K., Misra, A. K., Wig, N. N., & Susser, E. (2001). Mortality and long-term course in schizophrenia with a poor 2-year course: A study in a developing country. The British Journal of Psychiatry, 178(1), 71–75. 10.1192/bjp.178.1.71. 11136214

[r42] Moodley, L., Ntlantsana, V., Tomita, A., & Paruk, S. (2023). The missed pandemic: Intimate partner violence in female mental-health-care-users during the COVID-19 pandemic. Psychology Health & Medicine. 10.1080/13548506.2023.2206143. 37122135

[r43] Mpango, R. S., Ssembajjwe, W., Rukundo, G. Z., Amanyire, P., Birungi, C., Kalungi, A., Rutakumwa, R., Tusiime, C., Gadow, K. D., Patel, V., Nyirenda, M., & Kinyanda, E. (2023). Physical and sexual victimization of persons with severe mental illness seeking care in central and southwestern Uganda. Frontiers in Public Health, 11. 10.3389/fpubh.2023.1167076. PMC10446879 37621606

[r44] Murthy, R. S., & Lakshminarayana, R. (2006). Mental health consequences of war: A brief review of research findings. World Psychiatry, 5(1), 25–30. 16757987 PMC1472271

[r45] Nair, S., Satyanarayana, V. A., & Desai, G. (2020). Prevalence and clinical correlates of intimate partner violence (IPV) in women with severe mental illness (SMI). Asian Journal of Psychiatry, 52. 10.1016/j.ajp.2020.102131. 32371366

[r46] Ng, L. C., Medhin, G., Hanlon, C., & Fekadu, A. (2019). Trauma exposure, depression, suicidal ideation, and alcohol use in people with severe mental disorder in Ethiopia. Social Psychiatry and Psychiatric Epidemiology, 54(7), 835–842. 10.1007/s00127-019-01673-2. 30788553 PMC7343339

[r47] OHCHR (2016) CRPD/C/UGA/CO/1: Concluding observations on the initial report of Uganda. *United Nations Office of the High Commissioner for Human Rights.* https://www.ohchr.org/en/documents/concluding-observations/crpdcugaco1-concluding-observations-initial-report-uganda

[r48] Opekitan, T., Emedoh, T. C., Ogunsemi, O. O., & Adegohun, A. A. (2017). Socio-demographic characteristics, partner characteristics, socioeconomic variables, and intimate partner violence in women with schizophrenia in south-South Nigeria. Journal of Health Care for the Poor and Underserved, 28(2), 707–720. 10.1353/hpu.2017.0069. 28529219

[r49] Paul, S. (2018). Are we doing enough? Stigma, discrimination and human rights violations of people living with schizophrenia in India: Implications for social work practice. Social Work in Mental Health, 16(2), 145–171. 10.1080/15332985.2017.1361887.

[r50] Peters, M. D. J., Marnie, C., Tricco, A. C., Pollock, D., Munn, Z., Alexander, L., McInerney, P., Godfrey, C. M., & Khalil, H. (2021). Updated methodological guidance for the conduct of scoping reviews. JBI Evidence Implementation, 19(1), 3–10. 10.1097/xeb.0000000000000277. 33570328

[r51] Ponnudurai, R., Jayakar, J., & Sathiya Sekaran, B. W. (2006). Assessment of mortality and marital status of schizophrenic patients over a period of 13 years. Indian Journal of Psychiatry, 48(2), 84–87. 10.4103/0019-5545.31595. 20703391 PMC2913571

[r52] Ran, M. S., Chen, E. Y., Conwell, Y., Chan, C. L., Yip, P. S., Xiang, M. Z., & Caine E. D. (2007). Mortality in people with schizophrenia in rural China: 10-year cohort study. The British Journal of Psychiatry, 190, 237–242. 10.1192/bjp.bp.106.025155. 17329744

[r53] Rani, A., Raman, K. J., Antony, S., Thirumoorthy, A., & Basavarajappa, C. (2023). Profiles of victimized outpatients with severe mental illness in India. Indian Journal of Community Medicine, 48(6), 920–925. 10.4103/ijcm.ijcm_915_22. 38249707 PMC10795865

[r54] Rani, A., Raman, K. J., Antony, S., Thirumoorthy, A., & Chethan, B. (2023). A qualitative study to understand the nature of abuse experienced by persons with severe mental illness. Violence and Gender. 10.1089/vio.2022.0046.

[r55] Read, J., van Os, J., Morrison, A. P., & Ross, C. A. (2005). Childhood trauma, psychosis and schizophrenia: A literature review with theoretical and clinical implications. Acta Psychiatrica Scandinavica, 112(5), 330–350. 10.1111/j.1600-0447.2005.00634.x. 16223421

[r56] Read, U. M., Adiibokah, E., & Nyame, S. (2009). Local suffering and the global discourse of mental health and human rights: An ethnographic study of responses to mental illness in rural Ghana. Globalization and Health, 5, 13. 10.1186/1744-8603-5-13. 19825191 PMC2770454

[r57] Reddy, P. V., Tansa, K. A., Raj, A., Jangam, K., & Muralidharan, K. (2020). Childhood abuse and intimate partner violence among women with mood disorders. Journal of Affective Disorders, 272, 335–339. 10.1016/j.jad.2020.03.113. 32553375

[r58] Sadath, A., Narasimha, V. M., Rao, M., Kumar, V., Muralidhar, D., Varambally, S., Gangadhar, B. N., Kori, A., & Supraja, A. (2014). Human rights violation in mental health: A case report from India. African Journal of Psychiatry, 17(3), 1–2. http://ezp-prod1.hul.harvard.edu/login?url=https://search.ebscohost.com/login.aspx?direct=true&db=psyh&AN=2014-36342-006&site=ehost-live&scope=siteani00711@yahoo.com

[r59] Sam, S. P., Nisha, A., & Varghese, P. J. (2019). Stressful life events and relapse in bipolar affective disorder: A cross-sectional study from a tertiary Care Center of Southern India. Indian Journal of Psychology Medicine, 41(1), 61–67. 10.4103/IJPSYM.IJPSYM_113_18. PMC6337920 30783310

[r60] Subramanian, K., Sarkar, S., Kattimani, S., Philip Rajkumar, R., & Penchilaiya, V. (2017). Role of stressful life events and kindling in bipolar disorder: Converging evidence from a mania-predominant illness course. Psychiatry Research, 258, 434–437. 10.1016/j.psychres.2017.08.073. 28870645

[r61] Suryani, L. K., Lesmana, C. B. J., & Tiliopoulos, N. (2011). Treating the untreated: Applying a community-based, culturally sensitive psychiatric intervention to confined and physically restrained mentally ill individuals in Bali, Indonesia [article]. European Archives of Psychiatry and Clinical Neuroscience, 261(SUPPL. 2), S140–S144. 10.1007/s00406-011-0238-y. 21863345

[r62] Teferra, S., Shibre, T., Fekadu, A., Medhin, G., Wakwoya, A., Alem, A., Kullgren, G., & Jacobsson, L. (2011). Five-year mortality in a cohort of people with schizophrenia in Ethiopia. BMC Psychiatry, 11, 165. 10.1186/1471-244x-11-165. 21985179 PMC3207944

[r63] Tricco, A., Lillie, E., Zarin, W., O’Brien, K., Colquhoun, H., Levac, D., Moher, D., Peters, M., Horsley, T., Weeks, L., & Hempel, S. (2018). PRISMA extension for scoping reviews (PRISMA-ScR): Checklist and explanation. Annals of Internal Medicine, 169(7), 467–473. 10.7326/m18-0850. 30178033

[r64] Tsigebrhan, R., Shibre, T., Medhin, G., Fekadu, A., & Hanlon, C. (2014). Violence and violent victimization in people with severe mental illness in a rural low-income country setting: A comparative cross-sectional community study. Schizophrenia Research, 152(1), 275–282. 10.1016/j.schres.2013.10.032. 24275579

[r65] van de Schoot, R., de Bruin, J., Schram, R., Zahedi, P., de Boer, J., Weijdema, F., Kramer, B., Huijts, M., Hoogerwerf, M., Ferdinands, G., Harkema, A., Willemsen, J., Ma, Y., Fang, Q., Hindriks, S., Tummers, L., & Oberski, D. L. (2021). An open source machine learning framework for efficient and transparent systematic reviews. Nature Machine Intelligence, 3(2), 125–133. 10.1038/s42256-020-00287-7.

[r66] Varese, F., Smeets, F., Drukker, M., Lieverse, R., Lataster, T., Viechtbauer, W., Read, J., van Os, J., & Bentall, R. P. (2012). Childhood adversities increase the risk of psychosis: A meta-analysis of patient-control, prospective- and cross-sectional cohort studies. Schizophrenia Bulletin, 38(4), 661–671. 10.1093/schbul/sbs050. 22461484 PMC3406538

[r67] Veritas Health Innovation Ltd. (2024) *Covidence.* www.covidence.org

[r68] Vijayalakshmi, P., Reddemma, K., & Math, S. B. (2012). Gender-related differences in the human rights needs of patients with mental illness. Journal of Nursing Research, 20(2), 90–98. 10.1097/jnr.0b013e318257b57b. 22592104

[r69] Wang, Q. W., Wang, Q. W., Hou, C. L., Wang, S. B., Huang, Z. H., Huang, Y. H., Zhang, J. J., Zhang, J. J., & Jia, F. J. (2020). Frequency and correlates of violence against patients with schizophrenia living in rural China [Article]. BMC Psychiatry, 20(1). 10.1186/s12888-020-02696-9. PMC7275550 32505208

[r70] Weathers, F., Blake, D., Schnurr, P., Kaloupek, D., Marx, B., & Keane, T. (2013). The Life Events Checklist for DSM-5 (LEC-5) Standard. [Measurement Instrument.]. National Center for PTSD. https://www.ptsd.va.gov/professional/assessment/documents/LEC5_Standard_Self-report.PDF

[r71] Windarwati, H. D., Keliat, B. A., Ismail, R. I., Bachtiar, A., & Erawati, E. (2021). A journey of hidden outburst of anger shackling a person with schizophrenia: The Indonesian context. Qualitative Report, 26(8), 2577–2597. 10.46743/2160-3715/2021.4728.

[r72] Woolway, G. E., Smart, S. E., Lynham, A. J., Lloyd, J. L., Owen, M. J., Jones, I. R., Walters, J. T. R., & Legge, S. E. (2022). Schizophrenia polygenic risk and experiences of childhood adversity: A systematic review and meta-analysis. Schizophrenia Bulletin, 48(5), 967–980. 10.1093/schbul/sbac049. 35674151 PMC9434424

[r73] World Bank (2023) *World Bank Country and Lending Groups.* Retrieved 23 November 2023 from https://datahelpdesk.worldbank.org/knowledgebase/articles/906519-world-bank-country-and-lending-groups

[r74] Yosep, I., Mediani, H. S., Lindayani, L., & Sriati, A. (2021). How patients with schizophrenia “as a victim” cope with violence in Indonesia: A qualitative study. EGYPTIAN JOURNAL OF NEUROLOGY PSYCHIATRY AND NEUROSURGERY, 57(1). 10.1186/s41983-021-00327-y.

[r75] Yosep, I., Mediani, H. S., & Sriati, A. (2021). Experiences of violence among individuals with schizophrenia in Indonesia a phenomenological study. Journal of Psychosocial Nursing and Mental Health Services, 59(11), 41–46. 10.3928/02793695-20210528-01. 34251933

[r76] Zerihun, T., Tesfaye, M., Deyessa, N., & Bekele, D. (2021). Intimate partner violence among reproductive-age women with chronic mental illness attending a psychiatry outpatient department: Cross-sectional facility-based study, Addis Ababa, Ethiopia. BMJ Open, 11(12), e045251. 10.1136/bmjopen-2020-045251. PMC8655586 34880005

[r77] Zhang, Y. S., Li, K. Q., Sun, J. H., Li, W., Tong, Z. H., Yan, B. P., Ungvari, G. S., Ng, C. H., & Xiang, Y. T. (2018). Long-term outcomes of unlocking Chinese patients with severe mental illness [article]. Psychiatric Quarterly, 89(3), 757–763. 10.1007/s11126-018-9575-6. 29637466

[r78] Zhu, X. M., Xiang, Y. T., Zhou, J. S., Gou, L., Himelhoch, S., Ungvari, G. S., Chiu, H. F. K., Lai, K. Y. C., & Wang, X. P. (2014). Frequency of physical restraint and its associations with demographic and clinical characteristics in a Chinese psychiatric institution. Perspectives in Psychiatric Care, 50(4), 251–256. 10.1111/ppc.12049. 24308920

[r79] Zubin, J., & Spring, B. (1977). Vulnerability--a new view of schizophrenia. Journal of Abnormal Psychology, 86(2), 103–126. 10.1037//0021-843x.86.2.103. 858828

