# Supplementary material for: Serious mental health conditions and exposure to adulthood trauma in low- and middle-income countries: a scoping review
Source: Glob Ment Health (Camb). 2024 Nov 20;11:e112. doi: 10.1017/gmh.2024.123 (PMC11704373; doi:10.1017/gmh.2024.123)
Supplement: Stevenson et al. supplementary material [file S2054425124001237sup001.docx]

[**Africa-wide information/NiPAD**](https://hollis.harvard.edu/primo-explore/fulldisplay?docid=01HVD_ALMA612265994980003941&context=L&vid=HVD2&lang=en_US&search_scope=everything&adaptor=Local%20Search%20Engine&tab=everything&query=lsr01%2Ccontains%2C990088540570203941&mode=basic&offset=0)**:**

S1)

SU (Psychos#s OR Schizo* OR hallucinat* OR delusion* OR mania OR manic OR bipolar OR bi-polar OR "Serious mental illness" OR "Serious mental disorder" ) OR TI ( bipolar OR bi-polar OR delusion* OR mania OR manic OR psychotic OR psychos#s OR schizophren* OR schizoaffectiv* OR schizoid* OR (("mental disorder*" OR "mental illness*" OR "mental health" OR "psychiatric illness*" OR "psychiatric disorder*" OR "psychiatric condition*") N2 (severe OR serious))) OR AB ( bipolar OR bi-polar OR delusion* OR mania OR manic OR psychotic OR psychos#s OR OR schizophren* OR schizoaffectiv* OR schizoid* OR (("mental disorder*" OR "mental illness*" OR "mental health" OR "psychiatric illness*" OR "psychiatric disorder*" OR "psychiatric condition*") N2 (severe OR serious)))

S2)

SU ( "Emotional Trauma" OR "PTSD" OR "Post-Traumatic Stress" OR "Posttraumatic Stress Disorder" OR "Emotional Abuse" OR "Domestic Violence" OR "Intimate Partner Violence" OR "Physical Abuse" OR "Verbal Abuse" OR "Sexual Abuse" OR "Battered Females" OR "Rape" OR "Disasters" OR "Natural Disasters" OR "Incest" OR "Gun Violence" OR "War" OR "Parental Death" OR "Kidnapping" OR "Hostages" OR "Terrorism" OR "Torture" OR "injury") OR TI (abduct* OR accident* OR (acute N1 stress*) OR assault OR assaults OR assaulted OR atrocities OR ((battered OR beat*) N1 (person OR people OR wom#n OR m#n OR child* OR girl* OR boy* OR infant* OR wife OR wives OR spouse*)) OR captive* OR captivity OR "chemical war*" OR "chemical disaster*" OR "chemical spill*" OR (toxic* N2 expos*) OR combat OR cyclone* OR disaster OR disasters OR earthquake* OR ((abus* OR trauma*) N1 (emotional* OR physical* OR sexual* OR exposur* OR exposed OR event OR verbal* OR racial* OR psychological* OR social* OR child* OR sex)) OR (violence N1 (firearm* OR fire-arm* OR gun OR physical* OR gender OR ethnic* OR interpersonal* OR inter-personal* OR partner* OR mass OR psychological* OR racial* OR exposure OR exposed OR domestic OR school OR work-place OR workplace)) OR fight* OR fires OR floods OR drought* OR genocide OR harm OR "home invasion" OR homicide* OR hostage* OR hurricane* OR "human rights" OR incest* OR (idiom* N1 distress*) OR injur* OR kidnap* OR kid-nap* OR landslide* OR "life event*" OR LEC OR maltreat* OR molestation OR molested OR "mob justice" OR mugging OR mugged OR murder* OR "mass event*" OR post-traumatic OR posttraumatic OR rape OR raped OR rapes OR restrain* OR robber* OR "sexual coercion" OR shooting* OR stalk OR stalked OR stalking OR stalker* OR (Stressful N1 event*) OR "sudden death*" OR terroris* OR "tidal wave*" OR threaten* OR threat OR threats OR tornado* OR torture* OR "traumatic stress" OR "tropical storm*" OR tsunami* OR typhoon* OR victim* OR volcanic OR volcano* OR weapon* OR war OR wars OR warfare OR wildfire* OR wild-fire* OR injury OR injuries OR ((car OR road* OR traffic OR vehicle) N1 (collision* OR wreck* OR accident* OR pile-up))) OR AB (abduct* OR accident* OR (acute N1 stress*) OR assault OR assaults OR assaulted OR atrocities OR ((battered OR beat*) N1 (person OR people OR wom#n OR m#n OR child* OR girl* OR boy* OR infant* OR wife OR wives OR spouse*)) OR captive* OR captivity OR "chemical war*" OR "chemical disaster*" OR "chemical spill*" OR (toxic* N2 expos*) OR combat OR cyclone* OR disaster OR disasters OR earthquake* OR ((abus* OR trauma*) N1 (emotional* OR physical* OR sexual* OR exposur* OR exposed OR event OR verbal* OR racial* OR psychological* OR social* OR child* OR sex)) OR (violence N1 (firearm* OR fire-arm* OR gun OR physical* OR gender OR ethnic* OR interpersonal* OR inter-personal* OR partner* OR mass OR psychological* OR racial* OR exposure OR exposed)) OR fight* OR fires OR floods OR drought* OR genocide OR harm OR "home invasion" OR homicide* OR hostage* OR hurricane* OR "human rights" OR incest* OR (idiom* N1 distress*) OR injur* OR kidnap* OR kid-nap* OR landslide* OR "life event*" OR LEC OR maltreat* OR molestation OR molested OR "mob justice" OR mugging OR mugged OR murder* OR "mass event*" OR post-traumatic OR posttraumatic OR rape OR raped OR rapes OR restrain* OR robber* OR "sexual coercion" OR shooting* OR stalk OR stalked OR stalking OR stalker* OR (Stressful N1 event*) OR "sudden death*" OR terroris* OR "tidal wave*" OR threaten* OR threat OR threats OR tornado* OR torture* OR "traumatic stress" OR "tropical storm*" OR tsunami* OR typhoon* OR victim* OR volcanic OR volcano* OR weapon* OR war OR wars OR warfare OR wildfire* OR wild-fire* OR injury OR injuries OR ((car OR road* OR traffic OR vehicle) N1 (collision* OR wreck* OR accident* OR pile-up)))

S3)

ZG ("Africa" OR "Algeria" OR "Angola" OR "Benin" OR "Botswana" OR "Burkina Faso" OR "Burundi" OR "Cameroon" OR "Cape Verde" OR "Cabo Verde" OR "Central African Republic" OR "Chad" OR "Comoros" OR Congo OR "Côte d'Ivoire" OR "Ivory Coast" OR "Democratic Republic of the Congo" OR "Djibouti" OR "Egypt" OR "Equatorial Guinea" OR "Eritrea" OR "Ethiopia" OR "Gabon" OR "Gambia" OR "Ghana" OR "Guinea" OR "Guinea-Bissau" OR "Guinea Bissau" OR "Kenya" OR "Lesotho" OR "Liberia" OR "Libya" OR "Madagascar" OR "Malawi" OR "Mali" OR "Mauritania" OR "Mauritius" OR "Mayotte" OR "Morocco" OR "Mozambique" OR "Namibia" OR "Niger" OR "Nigeria" OR "Réunion" OR "Rwanda" OR "Sahara" OR "Sahel" OR "Sahel region" OR "Sao Tome and Principe" OR "Senegal" OR "Sierra Leone" OR "Somalia" OR "Somaliland" OR "Sub-Saharan Africa" OR "subsaharan africa" OR "Sudan" OR "Swaziland" OR "Togo" OR "Tunisia" OR "Uganda" OR "Tanzania" OR "Sahara" OR "Tanzania" OR "Zambia" OR "Zimbabwe" OR "East Africa" OR "east and west indian islands" OR "east europe" OR "eastern europe" OR "east asia" OR "Central Africa" OR "central America" OR "central asia" OR "Middle east" OR "North Africa" OR "maghreb" OR "Latin America" OR "Argentina" OR "Belize" OR "Bolivia" OR "Brazil" OR "Colombia" OR "Costa Rica" OR "Cuba" OR "Dominica" OR "Dominican Republic" OR "El Salvador" OR "Ecuador" OR "Grenada" OR "Guatemala" OR "Honduras" OR "Haiti" OR "Jamaica" OR "St. Lucia" OR "Saint Lucia" OR "Mexico" OR "Nicaragua" OR "Peru" OR "Paraguay" OR "Suriname" OR "St. Vincent" OR "Saint Vincent" OR "Venezuela" OR "South & Central America" OR "South America" OR "caribbean" OR "Southeast Asia" OR "southern & eastern asia" OR "Afghanistan" OR "Bangladesh" OR "Bhutan" OR "India" OR "Maldives" OR "Nepal" OR "Pakistan" OR "Sri Lanka" or "South Asia" OR "Iran" OR "Iraq" OR "Jordan" OR "Lebanon" OR "West Bank" OR "Gaza" OR "Palestine" OR "Palestinian Territories" OR "Syria" OR "Syrian Arab Republic" OR "Yemen" OR "Cambodia" OR "China" OR "Fiji" OR "Indonesia" OR "Kiribati" OR "Lao PDR" OR "Laos" OR "Malaysia" OR "Marshall Islands" OR "Micronesia" OR "Mongolia" OR "Myanmar" OR "Palau" OR "Papua New Guinea" OR "Philippines" OR "Samoa" OR "Solomon Islands" OR "Thailand" OR "Tonga" OR "Timor-Leste" OR "Vanuatu" OR "Vietnam" OR "Balkan States" OR "Albania" OR "Armenia" OR "Azerbaijan" OR "Belarus" OR "Bosnia" OR "Bulgaria" OR "Georgia" OR "Kazakhstan" OR "Kosovo" OR "Kosova" OR "Kyrgyzstan" OR "Moldova" OR "Montenegro" OR "North Macedonia" OR "Russia" OR "Russian Federation" OR "Serbia" OR "Tajikistan" OR "Turkmenistan" OR "Turkey" OR "Türkiye" OR "Ukraine" OR "Uzbekistan") OR TI (((Country OR countries) N1 (middle-income OR low-income OR low-middle OR developing OR low-GDP)) OR LMIC LMICs OR LAMIC OR LAMICs OR "global South" OR ((europe* OR africa* OR asia*) N1 east*) OR ((asia* OR africa*) N1 (central* OR north* OR west*)) OR (south* N1 asia*) OR (south*st N1 africa*) OR (america* N1 (south OR central OR latin)) OR Africa* OR Algeria* OR Angola* OR Benin OR Botswana OR "Burkina Faso" OR Burundi OR Cameroon* OR "Cape Verde" OR "Cabo verde" OR Chad OR Comoros OR comorian* OR Congo* OR "Côte d'Ivoire" OR "Ivory Coast" OR Djibouti* OR Egypt* OR Eritrea* OR Ethiopia* OR Gabon* OR Gambia* OR Ghana* OR Guinea* OR Kenya* OR Lesotho OR Liberia* OR Libya* OR Madagascar OR Malagasy OR Malawi* OR Mali* OR Mauritania* OR Mauritius OR Mayotte OR Morocc* OR Mozambique OR Namibia* OR Niger* OR Réunion OR Rwanda* OR saharan* OR sahel* OR "Sao Tome and Principe" OR Senegal* OR "Sierra Leone" OR Somali* OR Subsahara* OR Sudan* OR Swazi* OR Togo* OR Tunis* OR Uganda* OR Tanzania* OR Sahara* OR Zambia* OR Zimbabwe OR Argentin* OR Belize OR Bolivia* OR Bra#il* OR Colombia* OR "Costa Rica*" OR Cuba OR Cuban* OR Dominica* OR Salvador* OR Ecuador* OR Grenada OR Guatemala* OR Hondura* OR Haiti* OR Jamaica* OR "St. Lucia*" OR "Saint lucia*" OR Mexic* OR Nicaragua* OR Peru* OR Paraguay* OR Surinam* OR "St. Vincent" OR "Saint vincent* OR Venezuela* OR "Middle east*" OR Afghan* OR Bangladesh* OR bengali* OR Bhutan* OR India OR Maldives OR Nepal* OR Pakistan* OR "Sri Lanka*" OR Iran* OR Iraq* OR Jordan* OR Leban* OR "West Bank" OR Gaza OR gazan* OR Palestin* OR Syria* OR Yemen* OR Cambodia* OR China OR Chinese OR Fiji* OR Indonesia* OR Kiribati OR "Lao PDR" OR Laos OR laotian* OR Malaysia* OR "Marshall Islands" OR Micronesia* OR Mongolia* OR Myanmar OR Burma OR Burmese OR Palau OR Philippin* OR filipino OR Samoa* OR "Solomon Islands" OR Thailand OR Thai OR thais OR Tonga* OR Timor-Leste OR Vanuatu OR Vietnam* OR Albania* OR Armenia* OR Azerbaijan OR Azeri* OR Belarus* OR Bosnia* OR Bulgaria* OR Georgia OR Kazakh* OR Kosov* OR Kyrgyz* OR Moldova* OR Montenegr* OR macedonia* OR Russia* OR Serbia* OR Tajik* OR Turkmen* OR turk OR turks OR Turkey OR Türkiye OR Ukrain* OR Uzbek*) OR AB (((Country OR countries) N1 (middle-income OR low-income OR low-middle OR developing OR low-GDP)) OR LMIC LMICs OR LAMIC OR LAMICs OR "global South" OR ((europe* OR asia*) N1 east*) OR (asia* N1 (central* OR north* OR west* OR south*)) OR (america* N1 (south OR central OR latin)) OR Africa* OR Algeria* OR Angola* OR Benin OR Botswana OR "Burkina Faso" OR Burundi OR Cameroon* OR "Cape Verde" OR "Cabo verde" OR Chad OR Comoros OR comorian* OR Congo* OR "Côte d'Ivoire" OR "Ivory Coast" OR Djibouti* OR Egypt* OR Eritrea* OR Ethiopia* OR Gabon* OR Gambia* OR Ghana* OR Guinea* OR Kenya* OR Lesotho OR Liberia* OR Libya* OR Madagascar OR Malagasy OR Malawi* OR Mali* OR Mauritania* OR Mauritius OR Mayotte OR Morocc* OR Mozambique OR Namibia* OR Niger* OR Réunion OR Rwanda* OR saharan* OR sahel* OR "Sao Tome and Principe" OR Senegal* OR "Sierra Leone" OR Somali* OR Subsahara* OR Sudan* OR Swazi* OR Togo* OR Tunis* OR Uganda* OR Tanzania* OR Sahara* OR Zambia* OR Zimbabwe OR Argentin* OR Belize OR Bolivia* OR Bra#il* OR Colombia* OR "Costa Rica*" OR Cuba OR Cuban* OR Dominica* OR Salvador* OR Ecuador* OR Grenada OR Guatemala* OR Hondura* OR Haiti* OR Jamaica* OR "St. Lucia*" OR "Saint lucia*" OR Mexic* OR Nicaragua* OR Peru* OR Paraguay* OR Surinam* OR "St. Vincent" OR "Saint vincent* OR Venezuela* OR "Middle east*" OR Afghan* OR Bangladesh* OR bengali* OR Bhutan* OR India OR Maldives OR Nepal* OR Pakistan* OR "Sri Lanka*" OR Iran* OR Iraq* OR Jordan* OR Leban* OR "West Bank" OR Gaza OR gazan* OR Palestin* OR Syria* OR Yemen* OR Cambodia* OR China OR Chinese OR Fiji* OR Indonesia* OR Kiribati OR "Lao PDR" OR Laos OR laotian* OR Malaysia* OR "Marshall Islands" OR Micronesia* OR Mongolia* OR Myanmar OR Burma OR Burmese OR Palau OR Philippin* OR filipino OR Samoa* OR "Solomon Islands" OR Thailand OR Thai OR thais OR Tonga* OR Timor-Leste OR Vanuatu OR Vietnam* OR Albania* OR Armenia* OR Azerbaijan OR Azeri* OR Belarus* OR Bosnia* OR Bulgaria* OR Georgia OR Kazakh* OR Kosov* OR Kyrgyz* OR Moldova* OR Montenegr* OR macedonia* OR Russia* OR Serbia* OR Tajik* OR Turkmen* OR turk OR turks OR Turkey OR Türkiye OR Ukrain* OR Uzbek*) OR SU (((Country OR countries) N1 (middle-income OR low-income OR low-middle OR developing OR low-GDP)) OR LMIC LMICs OR LAMIC OR LAMICs OR "global South" OR ((europe* OR asia*) N1 east*) OR (asia* N1 (central* OR north* OR west* OR south*)) OR (america* N1 (south OR central OR latin)) OR africa* OR Algeria* OR Angola* OR Benin OR Botswana OR "Burkina Faso" OR Burundi OR Cameroon* OR "Cape Verde" OR "Cabo verde" OR Chad OR Comoros OR comorian* OR Congo* OR "Côte d'Ivoire" OR "Ivory Coast" OR Djibouti* OR Egypt* OR Eritrea* OR Ethiopia* OR Gabon* OR Gambia* OR Ghana* OR Guinea* OR Kenya* OR Lesotho OR Liberia* OR Libya* OR Madagascar OR Malagasy OR Malawi* OR Mali* OR Mauritania* OR Mauritius OR Mayotte OR Morocc* OR Mozambique OR Namibia* OR Niger* OR Réunion OR Rwanda* OR saharan* OR sahel* OR "Sao Tome and Principe" OR Senegal* OR "Sierra Leone" OR Somali* OR Subsahara* OR Sudan* OR Swazi* OR Togo* OR Tunis* OR Uganda* OR Tanzania* OR Sahara* OR Zambia* OR Zimbabwe OR Argentin* OR Belize OR Bolivia* OR Bra#il* OR Colombia* OR "Costa Rica*" OR Cuba OR Cuban* OR Dominica* OR Salvador* OR Ecuador* OR Grenada OR Guatemala* OR Hondura* OR Haiti* OR Jamaica* OR "St. Lucia*" OR "Saint lucia*" OR Mexic* OR Nicaragua* OR Peru* OR Paraguay* OR Surinam* OR "St. Vincent" OR "Saint vincent* OR Venezuela* OR "Middle east*" OR Afghan* OR Bangladesh* OR bengali* OR Bhutan* OR India OR Maldives OR Nepal* OR Pakistan* OR "Sri Lanka*" OR Iran* OR Iraq* OR Jordan* OR Leban* OR "West Bank" OR Gaza OR gazan* OR Palestin* OR Syria* OR Yemen* OR Cambodia* OR China OR Chinese OR Fiji* OR Indonesia* OR Kiribati OR "Lao PDR" OR Laos OR laotian* OR Malaysia* OR "Marshall Islands" OR Micronesia* OR Mongolia* OR Myanmar OR Burma OR Burmese OR Palau OR Philippin* OR filipino OR Samoa* OR "Solomon Islands" OR Thailand OR Thai OR thais OR Tonga* OR Timor-Leste OR Vanuatu OR Vietnam* OR Albania* OR Armenia* OR Azerbaijan OR Azeri* OR Belarus* OR Bosnia* OR Bulgaria* OR Georgia OR Kazakh* OR Kosov* OR Kyrgyz* OR Moldova* OR Montenegr* OR macedonia* OR Russia* OR Serbia* OR Tajik* OR Turkmen* OR turk OR turks OR Turkey OR Türkiye OR Ukrain* OR Uzbek*)

S4) S1 AND S2 AND S3

**PsycINFO:**

*Filter by:*

*age: adulthood*

*population group: human*

( DE “Psychosis” OR DE “Schizophrenia” OR DE “Acute Schizophrenia” OR DE “Catatonic Schizophrenia” OR DE “Schizoaffective Disorder” OR DE “Schizophrenia Disorganized Type” OR DE “Schizophreniform Disorder” OR DE “Undifferentiated Schizophrenia” OR DE “Chronic Psychosis” OR DE “Hallucinations” OR DE “Auditory Hallucinations” OR DE “Visual Hallucinations” OR DE “Affective Psychosis” OR DE “Mania” OR DE “Bipolar Disorder” ) OR TI ( bipolar OR delusion* OR mania OR “manic depress*” OR psychotic OR psychosis OR psychoses OR “serious mental disorder*” OR “serious mental illness” OR “severe mental disorder*” OR “severe mental illness” OR schizoaffective OR schizophren* OR “severe mental health condition*” ) OR DE ( bipolar OR delusion* OR mania OR “manic depress*” OR psychotic OR psychosis OR psychoses OR “serious mental disorder*” OR “serious mental illness” OR “severe mental disorder*” OR “severe mental illness” OR schizoaffective OR schizophren* OR “severe mental health condition*” ) OR AB ( bipolar OR delusion* OR mania OR “manic depress*” OR psychotic OR psychosis OR psychoses OR “serious mental disorder*” OR “serious mental illness” OR “severe mental disorder*” OR “severe mental illness” OR schizoaffective OR schizophren* OR “severe mental health condition*” ) OR KW ( bipolar OR delusion* OR mania OR “manic depress*” OR psychotic OR psychosis OR psychoses OR “serious mental disorder*” OR “serious mental illness” OR “severe mental disorder*” OR “severe mental illness” OR schizoaffective OR schizophren* OR “severe mental health condition*” )

AND

( DE “Emotional Trauma” OR DE “PTSD” OR DE “Post-Traumatic Stress” OR DE “Posttraumatic Stress Disorder” OR DE “Emotional Abuse” OR DE “Domestic Violence” OR DE “Intimate Partner Violence” OR DE “Physical Abuse” OR DE “Verbal Abuse” OR DE “Sexual Abuse” OR DE “Battered Females” OR DE “Rape*” OR DE “Disasters” OR DE “Natural Disasters” OR DE “Incest” OR DE “Gun Violence” OR DE “War” OR DE “Parental Death” OR DE “Kidnapping” OR DE “Hostages” OR DE “Terrorism” OR DE “Torture” ) OR TI ( abducted OR abduction OR accident* OR acute stress OR assault OR assaulted OR atrocities OR battered w* OR chain* OR captive OR captivity OR chemical OR “toxic substance” OR “toxic exposure” OR combat OR “combat disorder*” OR “combat neuros*” OR “combat stress disorder*” OR cyclone* OR disaster OR disasters OR earthquake* OR “emotional abuse” OR “emotional trauma*” OR “emotionally abus*” OR “ethnic violence” OR fight* OR “firearm violence” OR fires OR floods OR “gender-based violence” OR “gender violence” OR genocide OR “gun violence” OR harm OR “home invasion” OR homicide OR hostage* OR hurricane* OR “human rights” OR incest OR “idiom of distress” OR “idioms of distress” OR “interpersonal violence” OR injur* OR kidnap* OR landslides OR “life event”* OR LEC OR “life threaten*” OR maltreat* OR maltreatment OR molestation OR molested OR “mob justice” OR mug OR mugged OR murder OR “partner violence” OR “physical abuse” OR “physically abus*” OR “physical violence” OR “post traumatic” OR posttraumatic OR “psychological trauma” OR “racial violence” OR rape OR raped OR restrain* OR robber* OR “sexual abuse” OR “sexual coercion” OR “sexual violence” OR “sexually abused” OR shooting* OR stalk OR stalked OR “stressful event*” OR “stressful life event*” OR “sudden death” OR terroris* OR “tidal wave*” OR threaten* OR tornado* OR torture* OR “traumatic event*” OR “traumatic exposure*” OR “traumatic stress” OR “tropical storm*” OR tsunami* OR typhoon* OR victim* OR “verbal abuse” OR “verbally abused” OR volcanic OR volcano* OR weapon* OR wife beat* OR war OR warfare OR wildfires OR violent OR violence OR car OR road OR traffic OR vehicle ) OR DE ( abducted OR abduction OR accident* OR acute stress OR assault OR assaulted OR atrocities OR battered w* OR chain* OR captive OR captivity OR chemical OR “toxic substance” OR “toxic exposure” OR combat OR “combat disorder*” OR “combat neuros*” OR “combat stress disorder*” OR cyclone* OR disaster OR disasters OR earthquake* OR “emotional abuse” OR “emotional trauma*” OR “emotionally abus*” OR “ethnic violence” OR fight* OR “firearm violence” OR fires OR floods OR “gender-based violence” OR “gender violence” OR genocide OR “gun violence” OR harm OR “home invasion” OR homicide OR hostage* OR hurricane* OR “human rights” OR incest OR “idiom of distress” OR “idioms of distress” OR “interpersonal violence” OR injur* OR kidnap* OR landslides OR “life event”* OR LEC OR “life threaten*” OR maltreat* OR maltreatment OR molestation OR molested OR “mob justice” OR mug OR mugged OR murder OR “partner violence” OR “physical abuse” OR “physically abus*” OR “physical violence” OR “post traumatic” OR posttraumatic OR “psychological trauma” OR “racial violence” OR rape OR raped OR restrain* OR robber* OR “sexual abuse” OR “sexual coercion” OR “sexual violence” OR “sexually abused” OR shooting* OR stalk OR stalked OR “stressful event*” OR “stressful life event*” OR “sudden death” OR terroris* OR “tidal wave*” OR threaten* OR tornado* OR torture* OR “traumatic event*” OR “traumatic exposure*” OR “traumatic stress” OR “tropical storm*” OR tsunami* OR typhoon* OR victim* OR “verbal abuse” OR “verbally abused” OR volcanic OR volcano* OR weapon* OR wife beat* OR war OR warfare OR wildfires OR violent OR violence OR car OR road OR traffic OR vehicle ) OR AB ( abducted OR abduction OR accident* OR acute stress OR assault OR assaulted OR atrocities OR battered w* OR chain* OR captive OR captivity OR chemical OR “toxic substance” OR “toxic exposure” OR combat OR “combat disorder*” OR “combat neuros*” OR “combat stress disorder*” OR cyclone* OR disaster OR disasters OR earthquake* OR “emotional abuse” OR “emotional trauma*” OR “emotionally abus*” OR “ethnic violence” OR fight* OR “firearm violence” OR fires OR floods OR “gender-based violence” OR “gender violence” OR genocide OR “gun violence” OR harm OR “home invasion” OR homicide OR hostage* OR hurricane* OR “human rights” OR incest OR “idiom of distress” OR “idioms of distress” OR “interpersonal violence” OR injur* OR kidnap* OR landslides OR “life event”* OR LEC OR “life threaten*” OR maltreat* OR maltreatment OR molestation OR molested OR “mob justice” OR mug OR mugged OR murder OR “partner violence” OR “physical abuse” OR “physically abus*” OR “physical violence” OR “post traumatic” OR posttraumatic OR “psychological trauma” OR “racial violence” OR rape OR raped OR restrain* OR robber* OR “sexual abuse” OR “sexual coercion” OR “sexual violence” OR “sexually abused” OR shooting* OR stalk OR stalked OR “stressful event*” OR “stressful life event*” OR “sudden death” OR terroris* OR “tidal wave*” OR threaten* OR tornado* OR torture* OR “traumatic event*” OR “traumatic exposure*” OR “traumatic stress” OR “tropical storm*” OR tsunami* OR typhoon* OR victim* OR “verbal abuse” OR “verbally abused” OR volcanic OR volcano* OR weapon* OR wife beat* OR war OR warfare OR wildfires OR violent OR violence OR car OR road OR traffic OR vehicle ) OR KW ( abducted OR abduction OR accident* OR acute stress OR assault OR assaulted OR atrocities OR battered w* OR chain* OR captive OR captivity OR chemical OR “toxic substance” OR “toxic exposure” OR combat OR “combat disorder*” OR “combat neuros*” OR “combat stress disorder*” OR cyclone* OR disaster OR disasters OR earthquake* OR “emotional abuse” OR “emotional trauma*” OR “emotionally abus*” OR “ethnic violence” OR fight* OR “firearm violence” OR fires OR floods OR “gender-based violence” OR “gender violence” OR genocide OR “gun violence” OR harm OR “home invasion” OR homicide OR hostage* OR hurricane* OR “human rights” OR incest OR “idiom of distress” OR “idioms of distress” OR “interpersonal violence” OR injur* OR kidnap* OR landslides OR “life event”* OR LEC OR “life threaten*” OR maltreat* OR maltreatment OR molestation OR molested OR “mob justice” OR mug OR mugged OR murder OR “partner violence” OR “physical abuse” OR “physically abus*” OR “physical violence” OR “post traumatic” OR posttraumatic OR “psychological trauma” OR “racial violence” OR rape OR raped OR restrain* OR robber* OR “sexual abuse” OR “sexual coercion” OR “sexual violence” OR “sexually abused” OR shooting* OR stalk OR stalked OR “stressful event*” OR “stressful life event*” OR “sudden death” OR terroris* OR “tidal wave*” OR threaten* OR tornado* OR torture* OR “traumatic event*” OR “traumatic exposure*” OR “traumatic stress” OR “tropical storm*” OR tsunami* OR typhoon* OR victim* OR “verbal abuse” OR “verbally abused” OR volcanic OR volcano* OR weapon* OR wife beat* OR war OR warfare OR wildfires OR violent OR violence OR car OR road OR traffic OR vehicle )

AND

TI ( Africa OR Algeria OR Angola OR Benin OR Botswana OR “Burkina Faso” OR Burundi OR Cameroon OR Cape Verde OR “Central African Republic” OR Chad OR Comoros OR Congo OR “Côte d'Ivoire” OR “Ivory Coast” OR “Democratic Republic of the Congo” OR Djibouti OR Egypt OR “Equatorial Guinea” OR Eritrea OR Ethiopia OR Gabon OR Gambia OR Ghana OR Guinea OR Guinea-Bissau OR Kenya OR Lesotho OR Liberia OR Libya OR Madagascar OR Malawi OR Mali OR Mauritania OR Mauritius OR Mayotte OR Morocco OR Mozambique OR Namibia OR Niger OR Nigeria OR Réunion OR Rwanda OR “Sao Tome and Principe” OR Senegal OR “Sierra Leone” OR Somalia OR Somaliland OR “Sub-Saharan Africa” OR Sudan OR Swaziland OR Togo OR Tunisia OR Uganda OR Tanzania OR Sahara OR Tanzania OR Zambia OR Zimbabwe OR LMIC OR LMICs OR LAMIC OR “middle-income country” OR “middle-income countries” OR “low-income country” OR “low-income countries” OR “East Africa” OR “developing country” OR “developing countries” OR “Global South” low-resource*” OR Argentina OR Belize OR Bolivia OR Brazil OR Colombia OR “Costa Rica” OR Cuba OR Dominica OR “Dominican Republic” OR “El Salvador” OR Ecuador OR Grenada OR Guatemala OR Honduras OR Haiti OR Jamaica OR “St. Lucia” OR Mexico OR Nicaragua OR Peru OR Paraguay OR Suriname OR “St. Vincent” OR Venezuela OR “Latin America” OR “Central America OR “South America” OR Afghanistan OR Bangladesh OR Bhutan OR India OR Maldives OR Nepal OR Pakistan OR “Sri Lanka” or “South Asia” OR Iran OR Iraq OR Jordan OR Lebanon OR “West Bank” OR Gaza OR Palestine OR “Palestinian Territories” OR Syria OR “Syrian Arab Republic” OR Yemen OR Cambodia OR China OR Fiji OR Indonesia OR Kiribati OR “Lao PDR” OR Laos OR Malaysia OR “Marshall Islands” OR Micronesia OR Mongolia OR Myanmar OR Palau OR “Papua New Guinea” OR Philippines OR Samoa OR “Solomon Islands” OR Thailand OR Tonga OR Timor-Leste OR Vanuatu OR Vietnam OR Albania OR Armenia OR Azerbaijan OR Belarus OR Bosnia OR Bulgaria OR Georgia OR Kazakhstan OR Kosovo OR Kyrgyz OR Moldova OR Montenegro OR “North Macedonia” OR Russia OR “Russian Federation” OR Serbia OR Tajikistan OR Turkmenistan OR Turkey OR Türkiye OR Ukraine OR Uzbekistan OR “Eastern Europe*” OR “Central Asia*” ) OR SU ( Africa OR Algeria OR Angola OR Benin OR Botswana OR “Burkina Faso” OR Burundi OR Cameroon OR Cape Verde OR “Central African Republic” OR Chad OR Comoros OR Congo OR “Côte d'Ivoire” OR “Ivory Coast” OR “Democratic Republic of the Congo” OR Djibouti OR Egypt OR “Equatorial Guinea” OR Eritrea OR Ethiopia OR Gabon OR Gambia OR Ghana OR Guinea OR Guinea-Bissau OR Kenya OR Lesotho OR Liberia OR Libya OR Madagascar OR Malawi OR Mali OR Mauritania OR Mauritius OR Mayotte OR Morocco OR Mozambique OR Namibia OR Niger OR Nigeria OR Réunion OR Rwanda OR “Sao Tome and Principe” OR Senegal OR “Sierra Leone” OR Somalia OR Somaliland OR “Sub-Saharan Africa” OR Sudan OR Swaziland OR Togo OR Tunisia OR Uganda OR Tanzania OR Sahara OR Tanzania OR Zambia OR Zimbabwe OR LMIC OR LMICs OR LAMIC OR “middle-income country” OR “middle-income countries” OR “low-income country” OR “low-income countries” OR “East Africa” OR “developing country” OR “developing countries” OR “Global South” low-resource*” OR Argentina OR Belize OR Bolivia OR Brazil OR Colombia OR “Costa Rica” OR Cuba OR Dominica OR “Dominican Republic” OR “El Salvador” OR Ecuador OR Grenada OR Guatemala OR Honduras OR Haiti OR Jamaica OR “St. Lucia” OR Mexico OR Nicaragua OR Peru OR Paraguay OR Suriname OR “St. Vincent” OR Venezuela OR “Latin America” OR “Central America OR “South America” OR Afghanistan OR Bangladesh OR Bhutan OR India OR Maldives OR Nepal OR Pakistan OR “Sri Lanka” or “South Asia” OR Iran OR Iraq OR Jordan OR Lebanon OR “West Bank” OR Gaza OR Palestine OR “Palestinian Territories” OR Syria OR “Syrian Arab Republic” OR Yemen OR Cambodia OR China OR Fiji OR Indonesia OR Kiribati OR “Lao PDR” OR Laos OR Malaysia OR “Marshall Islands” OR Micronesia OR Mongolia OR Myanmar OR Palau OR “Papua New Guinea” OR Philippines OR Samoa OR “Solomon Islands” OR Thailand OR Tonga OR Timor-Leste OR Vanuatu OR Vietnam OR Albania OR Armenia OR Azerbaijan OR Belarus OR Bosnia OR Bulgaria OR Georgia OR Kazakhstan OR Kosovo OR Kyrgyz OR Moldova OR Montenegro OR “North Macedonia” OR Russia OR “Russian Federation” OR Serbia OR Tajikistan OR Turkmenistan OR Turkey OR Türkiye OR Ukraine OR Uzbekistan OR “Eastern Europe*” OR “Central Asia*” ) OR AB ( Africa OR Algeria OR Angola OR Benin OR Botswana OR “Burkina Faso” OR Burundi OR Cameroon OR Cape Verde OR “Central African Republic” OR Chad OR Comoros OR Congo OR “Côte d'Ivoire” OR “Ivory Coast” OR “Democratic Republic of the Congo” OR Djibouti OR Egypt OR “Equatorial Guinea” OR Eritrea OR Ethiopia OR Gabon OR Gambia OR Ghana OR Guinea OR Guinea-Bissau OR Kenya OR Lesotho OR Liberia OR Libya OR Madagascar OR Malawi OR Mali OR Mauritania OR Mauritius OR Mayotte OR Morocco OR Mozambique OR Namibia OR Niger OR Nigeria OR Réunion OR Rwanda OR “Sao Tome and Principe” OR Senegal OR “Sierra Leone” OR Somalia OR Somaliland OR “Sub-Saharan Africa” OR Sudan OR Swaziland OR Togo OR Tunisia OR Uganda OR Tanzania OR Sahara OR Tanzania OR Zambia OR Zimbabwe OR LMIC OR LMICs OR LAMIC OR “middle-income country” OR “middle-income countries” OR “low-income country” OR “low-income countries” OR “East Africa” OR “developing country” OR “developing countries” OR “Global South” low-resource*” OR Argentina OR Belize OR Bolivia OR Brazil OR Colombia OR “Costa Rica” OR Cuba OR Dominica OR “Dominican Republic” OR “El Salvador” OR Ecuador OR Grenada OR Guatemala OR Honduras OR Haiti OR Jamaica OR “St. Lucia” OR Mexico OR Nicaragua OR Peru OR Paraguay OR Suriname OR “St. Vincent” OR Venezuela OR “Latin America” OR “Central America OR “South America” OR Afghanistan OR Bangladesh OR Bhutan OR India OR Maldives OR Nepal OR Pakistan OR “Sri Lanka” or “South Asia” OR Iran OR Iraq OR Jordan OR Lebanon OR “West Bank” OR Gaza OR Palestine OR “Palestinian Territories” OR Syria OR “Syrian Arab Republic” OR Yemen OR Cambodia OR China OR Fiji OR Indonesia OR Kiribati OR “Lao PDR” OR Laos OR Malaysia OR “Marshall Islands” OR Micronesia OR Mongolia OR Myanmar OR Palau OR “Papua New Guinea” OR Philippines OR Samoa OR “Solomon Islands” OR Thailand OR Tonga OR Timor-Leste OR Vanuatu OR Vietnam OR Albania OR Armenia OR Azerbaijan OR Belarus OR Bosnia OR Bulgaria OR Georgia OR Kazakhstan OR Kosovo OR Kyrgyz OR Moldova OR Montenegro OR “North Macedonia” OR Russia OR “Russian Federation” OR Serbia OR Tajikistan OR Turkmenistan OR Turkey OR Türkiye OR Ukraine OR Uzbekistan OR “Eastern Europe*” OR “Central Asia*” ) OR KW ( Africa OR Algeria OR Angola OR Benin OR Botswana OR “Burkina Faso” OR Burundi OR Cameroon OR Cape Verde OR “Central African Republic” OR Chad OR Comoros OR Congo OR “Côte d'Ivoire” OR “Ivory Coast” OR “Democratic Republic of the Congo” OR Djibouti OR Egypt OR “Equatorial Guinea” OR Eritrea OR Ethiopia OR Gabon OR Gambia OR Ghana OR Guinea OR Guinea-Bissau OR Kenya OR Lesotho OR Liberia OR Libya OR Madagascar OR Malawi OR Mali OR Mauritania OR Mauritius OR Mayotte OR Morocco OR Mozambique OR Namibia OR Niger OR Nigeria OR Réunion OR Rwanda OR “Sao Tome and Principe” OR Senegal OR “Sierra Leone” OR Somalia OR Somaliland OR “Sub-Saharan Africa” OR Sudan OR Swaziland OR Togo OR Tunisia OR Uganda OR Tanzania OR Sahara OR Tanzania OR Zambia OR Zimbabwe OR LMIC OR LMICs OR LAMIC OR “middle-income country” OR “middle-income countries” OR “low-income country” OR “low-income countries” OR “East Africa” OR “developing country” OR “developing countries” OR “Global South” low-resource*” OR Argentina OR Belize OR Bolivia OR Brazil OR Colombia OR “Costa Rica” OR Cuba OR Dominica OR “Dominican Republic” OR “El Salvador” OR Ecuador OR Grenada OR Guatemala OR Honduras OR Haiti OR Jamaica OR “St. Lucia” OR Mexico OR Nicaragua OR Peru OR Paraguay OR Suriname OR “St. Vincent” OR Venezuela OR “Latin America” OR “Central America OR “South America” OR Afghanistan OR Bangladesh OR Bhutan OR India OR Maldives OR Nepal OR Pakistan OR “Sri Lanka” or “South Asia” OR Iran OR Iraq OR Jordan OR Lebanon OR “West Bank” OR Gaza OR Palestine OR “Palestinian Territories” OR Syria OR “Syrian Arab Republic” OR Yemen OR Cambodia OR China OR Fiji OR Indonesia OR Kiribati OR “Lao PDR” OR Laos OR Malaysia OR “Marshall Islands” OR Micronesia OR Mongolia OR Myanmar OR Palau OR “Papua New Guinea” OR Philippines OR Samoa OR “Solomon Islands” OR Thailand OR Tonga OR Timor-Leste OR Vanuatu OR Vietnam OR Albania OR Armenia OR Azerbaijan OR Belarus OR Bosnia OR Bulgaria OR Georgia OR Kazakhstan OR Kosovo OR Kyrgyz OR Moldova OR Montenegro OR “North Macedonia” OR Russia OR “Russian Federation” OR Serbia OR Tajikistan OR Turkmenistan OR Turkey OR Türkiye OR Ukraine OR Uzbekistan OR “Eastern Europe*” OR “Central Asia*” )

**Web of Science Core Collection:**

**(TS=((Schizophren* OR "Psychotic Disorders" OR “bipolar disorder” OR delusion* OR mania OR psychotic OR psychosis OR psychoses OR “serious mental illness” OR “severe mental illness” OR “serious mental disorder*” OR “severe mental disorder*” OR schizoaffective OR “severe mental health condition*”) ) AND TS=(("Life Change Event*" OR abducted OR abduction OR accident* OR “acute stress” OR assault OR assaulted OR atrocities OR battered OR chain* OR captive OR captivity OR chemical OR “toxic substance” OR “toxic exposure” OR combat OR “combat disorder*” OR “combat neuros*” OR “combat stress disorder*” OR cyclone* OR disaster* OR earthquake* OR “emotional abuse” OR “emotional trauma*” OR “emotionally abus*” OR “ethnic violence” OR fight* OR “firearm violence” OR fires OR floods OR “gender based violence” OR “gender violence” OR genocide OR “gun violence” OR harm OR “home invasion” OR homicide OR hostage* OR hurricane* OR “human rights” OR incest OR “idiom of distress” OR “idioms of distress” OR “interpersonal violence” OR injur* OR kidnap* OR landslides OR “life event*” OR LEC OR “life threaten*” OR maltreat* OR maltreatment OR molestation OR molested OR “mob justice” OR mug OR mugged OR murder OR “partner violence” OR “physical abuse” OR “physically abus*” OR “physical violence” OR “post traumatic” OR posttraumatic OR “psychological trauma” OR “racial violence” OR rape OR raped OR restrain* OR robber* OR “sexual abuse” OR “sexual coercion” OR “sexual violence” OR “sexually abused” OR shooting* OR stalk OR stalked OR “stressful event*” OR “stressful life event*” OR “sudden death” OR terroris* OR tidal wave* OR threaten* OR tornado* OR torture* OR “traumatic event*” OR “traumatic exposure*” OR “traumatic stress” OR “tropical storm*” OR tsunami* OR typhoon* OR victim* OR “verbal abuse” OR “verbally abused” OR volcanic OR volcano* OR weapon* OR “wife beat*” OR war OR warfare OR wildfires OR violent OR violence OR car OR road OR traffic OR vehicle)) AND TS=((Africa OR Algeria OR Angola OR Benin OR Botswana OR “Burkina Faso” OR Burundi OR Cameroon OR “Cape Verde” OR “Central African Republic” OR Chad OR Comoros OR Congo OR “Côte d'Ivoire” OR “Ivory Coast” OR “Democratic Republic of the Congo” OR Djibouti OR Egypt OR “Equatorial Guinea” OR Eritrea OR Ethiopia OR Gabon OR Gambia OR Ghana OR Guinea OR Guinea-Bissau OR Kenya OR Lesotho OR Liberia OR Libya OR Madagascar OR Malawi OR Mali OR Mauritania OR Mauritius OR Mayotte OR Morocco OR Mozambique OR Namibia OR Niger OR Nigeria OR Réunion OR Rwanda OR “Sao Tome and Principe” OR Senegal OR “Sierra Leone” OR Somalia OR Somaliland OR Sudan OR Swaziland OR Togo OR Tunisia OR Uganda OR Tanzania OR Sahara OR Tanzania OR Zambia OR Zimbabwe OR LMIC OR LMICs OR LAMIC OR “middle-income country” OR “middle-income countries” OR “low-income country” OR “low-income countries” OR “Sub-Saharan Africa” OR “East Africa” OR “developing country” OR “developing countries” OR “Global South” OR low-resource* OR Argentina OR Belize OR Bolivia OR Brazil OR Colombia OR “Costa Rica” OR Cuba OR Dominica OR “Dominican Republic” OR “El Salvador” OR Ecuador OR Grenada OR Guatemala OR Honduras OR Haiti OR Jamaica OR “St. Lucia” OR Mexico OR Nicaragua OR Peru OR Paraguay OR Suriname OR “St. Vincent” OR Venezuela OR Latin America OR Central America OR South America OR Afghanistan OR Bangladesh OR Bhutan OR India OR Maldives OR Nepal OR Pakistan OR “Sri Lanka” or “South Asia” OR Iran OR Iraq OR Jordan OR Lebanon OR “West Bank” OR Gaza OR Palestine or “Palestinian Territories” OR Syria OR “Syrian Arab Republic” OR Yemen OR Cambodia OR China OR Fiji OR Indonesia OR Kiribati OR “Lao PDR” OR Laos OR Malaysia OR “Marshall Islands” OR Micronesia OR Mongolia OR Myanmar OR Palau OR “Papua New Guinea” OR Philippines OR Samoa OR “Solomon Islands” OR Thailand OR Tonga OR Timor-Leste OR Vanuatu OR Vietnam OR Albania OR Armenia OR Azerbaijan OR Belarus OR Bosnia OR Bulgaria OR Georgia OR Kazakhstan OR Kosovo OR Kyrgyz OR Moldova OR Montenegro OR “North Macedonia” OR Russia OR “Russian Federation” OR Serbia OR Tajikistan OR Turkmenistan OR Turkey OR Türkiye OR Ukraine OR Uzbekistan OR “Eastern Europe*” OR Central Asia*)) NOT TS=((rat OR mouse OR animal OR “guinea pig” OR forensic OR comment* OR editorial OR letter))) AND (DT==("ARTICLE"))**

**Embase:**

('psychosis'/de OR 'schizophrenia'/de OR 'catatonic schizophrenia'/de OR 'paranoid schizophrenia'/de OR 'schizoaffective psychosis'/de OR 'affective psychosis'/de OR 'manic psychosis'/de OR 'bipolar disorder'/exp) NOT (rat OR mouse OR animal OR 'guinea pig' OR forensic OR 'case report'/de OR [conference abstract]/lim OR [conference paper]/lim OR [conference review]/lim OR [editorial]/lim OR [letter]/lim OR [note]/lim) AND [embase]/lim

('bipolar disorder':ab,ti OR delusion*:ab,ti OR mania:ab,ti OR 'manic depress*':ab,ti OR psychotic:ab,ti OR psychosis:ab,ti OR psychoses:ab,ti OR 'serious mental disorder*':ab,ti OR 'serious mental illness':ab,ti OR 'severe mental disorder*':ab,ti OR 'severe mental illness':ab,ti OR 'severe mental health condition*':ab,ti OR schizoaffective:ab,ti OR schizophren*:ab,ti) NOT (rat OR mouse OR animal OR 'guinea pig' OR forensic OR 'case report'/de OR [conference abstract]/lim OR [conference paper]/lim OR [conference review]/lim OR [editorial]/lim OR [letter]/lim OR [note]/lim) AND [embase]/lim

('assault'/de OR 'battered woman'/de OR 'battering'/de OR 'disaster'/de OR 'domestic violence'/exp OR 'earthquake'/de OR 'explosion'/de OR 'fire'/de OR 'gender based violence'/de OR 'ethnic conflict'/de OR 'genocide'/de OR 'hurricane'/de OR 'incest'/de OR 'kidnapping'/de OR 'landslide'/de OR 'mass disaster'/de OR 'natural disaster'/de OR 'parental death'/de OR 'physical abuse'/de OR 'physical violence'/de OR 'posttraumatic stress disorder'/de OR 'prisoner of war'/de OR 'sexual violence'/exp OR 'tornado'/de OR 'torture'/de OR 'torture survivor'/de OR 'tsunami'/de OR 'violence'/de OR 'volcano'/de OR 'war'/exp OR 'wildfire'/exp) NOT (rat OR mouse OR animal OR 'guinea pig' OR forensic OR 'case report'/de OR [conference abstract]/lim OR [conference paper]/lim OR [conference review]/lim OR [editorial]/lim OR [letter]/lim OR [note]/lim) AND [embase]/lim

((abducted:ab,ti OR abduction:ab,ti OR accident:ab,ti OR 'acute stress':ab,ti OR 'adult advers*':ab,ti OR 'adverse experience*':ab,ti OR assault:ab,ti OR assaulted:ab,ti OR atrocities:ab,ti OR 'battered w*':ab,ti OR captive:ab,ti OR captivity:ab,ti OR chain:ab,ti OR chemical:ab,ti OR 'combat disorder*':ab,ti OR 'combat neuros*':ab,ti OR 'combat stress disorder*':ab,ti OR cyclone*:ab,ti OR disaster:ab,ti OR disasters:ab,ti OR 'domestic violence':ab,ti OR earthquake*:ab,ti OR (((emotional* OR partner OR psychological* OR sexual*) NEAR/3 abus*):ab,ti) OR (((emotional* OR psychological*) NEXT/1 trauma*):ab,ti) OR 'ethnic conflict*':ab,ti OR 'ethnic violence':ab,ti OR (((expos* OR victim*) NEAR/3 violen*):ab,ti) OR fight*:ab,ti OR 'firearm violence':ab,ti OR fires:ab,ti OR floods:ab,ti OR 'gender based violence':ab,ti OR 'gender violence':ab,ti OR genocide:ab,ti OR 'gun violence':ab,ti OR harm:ab,ti OR 'home invasion':ab,ti OR homicide:ab,ti OR hostage*:ab,ti OR 'human rights':ab,ti OR hurricane*:ab,ti OR 'idiom of distress':ab,ti OR 'idioms of distress':ab,ti OR incest:ab,ti OR 'interpersonal violence':ab,ti OR injur*:ab,ti OR kidnap*:ab,ti OR landslides:ab,ti) AND 'life event*':ab,ti OR lec:ab,ti OR 'life threaten*':ab,ti OR maltreat*:ab,ti OR 'mob justice':ab,ti OR mug:ab,ti OR mugged:ab,ti OR murder:ab,ti OR molestation:ab,ti OR molested:ab,ti OR ((parent* NEAR/3 (loss OR death*)):ab,ti) OR 'partner violence':ab,ti OR 'physical abuse':ab,ti OR 'physically abused':ab,ti OR 'post traumatic':ab,ti OR posttraumatic:ab,ti OR 'racial violence':ab,ti OR rape:ab,ti OR raped:ab,ti OR restrain*:ab,ti OR robber*:ab,ti OR 'sexual coercion':ab,ti OR 'sexual violence':ab,ti OR shooting*:ab,ti OR stalk:ab,ti OR stalked:ab,ti OR 'stressful event*':ab,ti OR 'stressful life event*':ab,ti OR 'sudden death':ab,ti OR terroris*:ab,ti OR 'tidal wave*':ab,ti OR threaten*:ab,ti OR tornado*:ab,ti OR torture*:ab,ti OR 'toxic substance':ab,ti OR 'toxic exposure':ab,ti OR 'traumatic event*':ab,ti OR 'traumatic stress':ab,ti OR 'tropical storm*':ab,ti OR tsunami*:ab,ti OR typhoon*:ab,ti OR 'verbal abuse':ab,ti OR 'verbally abused':ab,ti OR violent:ab,ti OR violence:ab,ti OR volcanic:ab,ti OR volcano*:ab,ti OR war:ab,ti OR warfare:ab,ti OR car:ab,ti OR road:ab,ti OR traffic:ab,ti OR vehicle:ab,ti OR weapon*:ab,ti OR 'wife beat*':ab,ti OR wildfires:ab,ti) NOT (rat OR mouse OR animal OR 'guinea pig' OR forensic OR 'case report'/de OR [conference abstract]/lim OR [conference paper]/lim OR [conference review]/lim OR [editorial]/lim OR [letter]/lim OR [note]/lim) AND [embase]/lim

(((((africa:ab,ti OR algeria:ab,ti OR angola:ab,ti OR benin:ab,ti OR botswana:ab,ti OR burkina:ab,ti) AND faso:ab,ti OR burundi:ab,ti OR cameroon:ab,ti OR 'cape verde':ab,ti OR 'central african republic':ab,ti OR chad:ab,ti OR comoros:ab,ti OR congo:ab,ti OR 'cote divoire':ab,ti OR 'ivory coast':ab,ti OR democratic:ab,ti) AND republic:ab,ti AND of:ab,ti AND the:ab,ti AND congo:ab,ti OR djibouti:ab,ti OR egypt:ab,ti OR 'equatorial guinea':ab,ti OR eritrea:ab,ti OR ethiopia:ab,ti OR gabon:ab,ti OR gambia:ab,ti OR ghana:ab,ti OR guinea:ab,ti OR 'guinea bissau':ab,ti OR kenya:ab,ti OR lesotho:ab,ti OR liberia:ab,ti OR libya:ab,ti OR madagascar:ab,ti OR malawi:ab,ti OR mali:ab,ti OR mauritania:ab,ti OR mauritius:ab,ti OR mayotte:ab,ti OR morocco:ab,ti OR mozambique:ab,ti OR namibia:ab,ti OR niger:ab,ti OR nigeria:ab,ti OR réunion:ab,ti OR rwanda:ab,ti OR 'sao tome and principe':ab,ti OR senegal:ab,ti OR 'sierra leone':ab,ti OR somalia:ab,ti OR somaliland:ab,ti OR sudan:ab,ti OR swaziland:ab,ti OR togo:ab,ti OR tunisia:ab,ti OR uganda:ab,ti OR sahara:ab,ti OR tanzania:ab,ti OR zambia:ab,ti OR zimbabwe:ab,ti OR lmic:ab,ti OR lmics:ab,ti OR lamic:ab,ti OR 'middle-income country':ab,ti OR 'middle-income countries':ab,ti OR 'low-income country':ab,ti OR 'low-income countries':ab,ti OR 'sub-saharan africa':ab,ti OR 'east africa':ab,ti OR 'developing country':ab,ti OR 'developing countries':ab,ti OR 'global south':ab,ti OR 'low resource*':ab,ti OR argentina:ab,ti OR belize:ab,ti OR bolivia:ab,ti OR brazil:ab,ti OR colombia:ab,ti OR 'costa rica':ab,ti OR cuba:ab,ti OR dominica:ab,ti OR 'dominican republic':ab,ti OR 'el salvador':ab,ti OR ecuador:ab,ti OR grenada:ab,ti OR guatemala:ab,ti OR honduras:ab,ti OR haiti:ab,ti OR jamaica:ab,ti OR 'st. lucia':ab,ti OR mexico:ab,ti OR nicaragua:ab,ti OR peru:ab,ti OR paraguay:ab,ti OR suriname:ab,ti OR 'st. vincent':ab,ti OR venezuela:ab,ti OR 'latin america':ab,ti OR 'central america':ab,ti OR 'south america':ab,ti OR afghanistan:ab,ti OR bangladesh:ab,ti OR bhutan:ab,ti OR india:ab,ti OR maldives:ab,ti OR nepal:ab,ti OR pakistan:ab,ti OR sri:ab,ti) AND lanka:ab,ti OR south:ab,ti) AND asia:ab,ti OR iran:ab,ti OR iraq:ab,ti OR jordan:ab,ti OR lebanon:ab,ti OR 'west bank':ab,ti OR gaza:ab,ti OR palestine:ab,ti OR 'palestinian territories':ab,ti OR syria:ab,ti OR 'syrian arab republic':ab,ti OR yemen:ab,ti OR cambodia:ab,ti OR china:ab,ti OR fiji:ab,ti OR indonesia:ab,ti OR kiribati:ab,ti OR 'lao pdr':ab,ti OR laos:ab,ti OR malaysia:ab,ti OR 'marshall islands':ab,ti OR micronesia:ab,ti OR mongolia:ab,ti OR myanmar:ab,ti OR palau:ab,ti OR 'papua new guinea':ab,ti OR philippines:ab,ti OR samoa:ab,ti OR 'solomon islands':ab,ti OR thailand:ab,ti OR tonga:ab,ti OR 'timor leste':ab,ti OR vanuatu:ab,ti OR vietnam:ab,ti OR albania:ab,ti OR armenia:ab,ti OR azerbaijan:ab,ti OR belarus:ab,ti OR bosnia:ab,ti OR bulgaria:ab,ti OR georgia:ab,ti OR kazakhstan:ab,ti OR kosovo:ab,ti OR kyrgyz:ab,ti OR moldova:ab,ti OR montenegro:ab,ti OR 'north macedonia':ab,ti OR russia:ab,ti OR 'russian federation':ab,ti OR serbia:ab,ti OR tajikistan:ab,ti OR turkmenistan:ab,ti OR turkey:ab,ti OR türkiye:ab,ti OR ukraine:ab,ti OR uzbekistan:ab,ti OR 'eastern europe*':ab,ti OR 'central asia*':ab,ti) NOT (rat OR mouse OR animal OR 'guinea pig' OR forensic OR 'case report'/de OR [conference abstract]/lim OR [conference paper]/lim OR [conference review]/lim OR [editorial]/lim OR [letter]/lim OR [note]/lim) AND [embase]/lim

**PubMed:**

((((("Schizophrenia"[mh] OR "Psychotic Disorders"[mh:noexp] OR bipolar[tiab] OR delusion*[tiab] OR mania[tiab] OR psychotic[tiab] OR psychosis[tiab] OR psychoses[tiab] OR serious mental illness[tiab] OR serious mental disorder*[tiab] OR severe mental disorder*[tiab] OR schizoaffective[tiab] OR schizophren*[tiab] OR severe mental health condition*[tiab])) AND (("Partner Violence"[tiab] OR "Physical Abuse"[mh] OR "Rape"[mh] OR "Incest"[mh] OR "Life Change Events"[mh] OR "Warfare"[mh] OR "Terrorism"[mh:noexp] OR "Torture"[mh] OR "Disasters"[mh:noexp] OR "Natural Disasters"[mh] OR "Tsunamis"[mh] OR "Volcanic Eruptions"[mh] OR "Fires"[mh:noexp] OR abducted[tiab] OR abduction[tiab] OR accident*[tiab] OR acute stress[tiab] OR assault[tiab] OR assaulted[tiab] OR atrocities[tiab] OR battered w*[tiab] OR chain*[tiab] OR captive[tiab] OR captivity[tiab] OR chemical[tiab] OR toxic substance[tiab] OR toxic exposure[tiab] OR combat[tiab] OR combat disorder*[tiab] OR combat neuros*[tiab] OR combat stress disorder*[tiab] OR cyclone*[tiab] OR disaster[tiab] OR disasters[tiab] OR earthquake*[tiab] OR emotional abuse[tiab] OR emotional trauma*[tiab] OR emotionally abus*[tiab] OR ethnic violence[tiab] OR fight*[tiab] OR firearm violence[tiab] OR fires[tiab] OR floods[tiab] OR gender based violence[tiab] OR gender violence[tiab] OR genocide[tiab] OR gun violence[tiab] OR harm[tiab] OR home invasion[tiab] OR homicide[tiab] OR hostage*[tiab] OR hurricane*[tiab] OR human rights[tiab] OR incest[tiab] OR idiom of distress[tiab] OR idioms of distress[tiab] OR interpersonal violence[tiab] OR injur*[tiab] OR kidnap*[tiab] OR landslides[tiab] OR life event*[tiab] OR LEC[tiab] OR life threaten*[tiab] OR maltreat*[tiab] OR maltreatment[tiab] OR molestation[tiab] OR molested[tiab] OR mob justice[tiab] OR mug[tiab] OR mugged[tiab] OR murder[tiab] OR partner violence[tiab] OR physical abuse[tiab] OR physically abus*[tiab] OR physical violence[tiab] OR post traumatic[tiab] OR posttraumatic[tiab] OR psychological trauma[tiab] OR racial violence[tiab] OR rape[tiab] OR raped[tiab] OR restrain*[tiab] OR robber*[tiab] OR sexual abuse[tiab] OR sexual coercion[tiab] OR sexual violence[tiab] OR sexually abused[tiab] OR shooting*[tiab] OR stalk[tiab] OR stalked[tiab] OR stressful event*[tiab] OR stressful life event*[tiab] OR sudden death[tiab] OR terroris*[tiab] OR tidal wave*[tiab] OR threaten*[tiab] OR tornado*[tiab] OR torture*[tiab] OR traumatic event*[tiab] OR traumatic exposure*[tiab] OR traumatic stress[tiab] OR tropical storm*[tiab] OR tsunami*[tiab] OR typhoon*[tiab] OR victim*[tiab] OR verbal abuse[tiab] OR verbally abused[tiab] OR volcanic[tiab] OR volcano*[tiab] OR weapon*[tiab] OR wife beat*[tiab] OR war[tiab] OR warfare[tiab] OR wildfires[tiab] OR violent[tiab] OR violence[tiab] OR car[tiab] OR road[tiab] OR traffic[tiab] OR vehicle[tiab]))) AND ((Africa[tiab] OR Algeria[tiab] OR Angola[tiab] OR Benin[tiab] OR Botswana[tiab] OR Burkina Faso[tiab] OR Burundi[tiab] OR Cameroon[tiab] OR Cape Verde[tiab] OR Central African Republic[tiab] OR Chad[tiab] OR Comoros[tiab] OR Congo[tiab] OR Côte d'Ivoire[tiab] OR Ivory Coast[tiab] OR Democratic Republic of the Congo[tiab] OR Djibouti[tiab] OR Egypt[tiab] OR Equatorial Guinea[tiab] OR Eritrea[tiab] OR Ethiopia[tiab] OR Gabon[tiab] OR Gambia[tiab] OR Ghana[tiab] OR Guinea[tiab] OR Guinea-Bissau[tiab] OR Kenya[tiab] OR Lesotho[tiab] OR Liberia[tiab] OR Libya[tiab] OR Madagascar[tiab] OR Malawi[tiab] OR Mali[tiab] OR Mauritania[tiab] OR Mauritius[tiab] OR Mayotte[tiab] OR Morocco[tiab] OR Mozambique[tiab] OR Namibia[tiab] OR Niger[tiab] OR Nigeria[tiab] OR Réunion[tiab] OR Rwanda[tiab] OR Sao Tome and Principe[tiab] OR Senegal[tiab] OR Sierra Leone[tiab] OR Somalia[tiab] OR Somaliland[tiab] OR Sudan[tiab] OR Swaziland[tiab] OR Togo[tiab] OR Tunisia[tiab] OR Uganda[tiab] OR Tanzania[tiab] OR Sahara[tiab] OR Tanzania[tiab] OR Zambia[tiab] OR Zimbabwe[tiab] OR LMIC[tiab] OR LMICs[tiab] OR LAMIC[tiab] OR middle-income country[tiab] OR middle-income countries[tiab] OR low-income country[tiab] OR low-income countries[tiab] OR Sub-Saharan Africa[tiab] OR East Africa[tiab] OR developing country[tiab] OR developing countries[tiab] OR Global South[tiab] OR low-resource*[tiab] OR Argentina[tiab] OR Belize[tiab] OR Bolivia[tiab] OR Brazil[tiab] OR Colombia[tiab] OR Costa Rica[tiab] OR Cuba[tiab] OR Dominica[tiab] OR Dominican Republic[tiab] OR El Salvador[tiab] OR Ecuador[tiab] OR Grenada[tiab] OR Guatemala[tiab] OR Honduras[tiab] OR Haiti[tiab] OR Jamaica[tiab] OR St. Lucia[tiab] OR Mexico[tiab] OR Nicaragua[tiab] OR Peru[tiab] OR Paraguay[tiab] OR Suriname[tiab] OR St. Vincent[tiab] OR Venezuela[tiab] OR Latin America[tiab] OR Central America[tiab] OR South America[tiab] OR Afghanistan[tiab] OR Bangladesh[tiab] OR Bhutan[tiab] OR India[tiab] OR Maldives[tiab] OR Nepal[tiab] OR Pakistan[tiab] OR Sri Lanka[tiab] or South Asia[tiab] OR Iran[tiab] OR Iraq[tiab] OR Jordan[tiab] OR Lebanon[tiab] OR West Bank[tiab] OR Gaza[tiab] OR Palestine[tiab] or Palestinian Territories[tiab] OR Syria[tiab] OR Syrian Arab Republic[tiab] OR Yemen[tiab] OR Cambodia[tiab] OR China[tiab] OR Fiji[tiab] OR Indonesia[tiab] OR Kiribati[tiab] OR Lao PDR[tiab] OR Laos[tiab] OR Malaysia[tiab] OR Marshall Islands[tiab] OR Micronesia[tiab] OR Mongolia[tiab] OR Myanmar[tiab] OR Palau[tiab] OR Papua New Guinea[tiab] OR Philippines[tiab] OR Samoa[tiab] OR Solomon Islands[tiab] OR Thailand[tiab] OR Tonga[tiab] OR Timor-Leste[tiab] OR Vanuatu[tiab] OR Vietnam[tiab] OR Albania[tiab] OR Armenia[tiab] OR Azerbaijan[tiab] OR Belarus[tiab] OR Bosnia[tiab] OR Bulgaria[tiab] OR Georgia[tiab] OR Kazakhstan[tiab] OR Kosovo[tiab] OR Kyrgyz[tiab] OR Moldova[tiab] OR Montenegro[tiab] OR North Macedonia[tiab] OR Russia[tiab] OR Russian Federation[tiab] OR Serbia[tiab] OR Tajikistan[tiab] OR Turkmenistan[tiab] OR Turkey[tiab] OR Türkiye[tiab] OR Ukraine[tiab] OR Uzbekistan[tiab] OR Eastern Europe*[tiab] OR Central Asia*[tiab]))) NOT ((Comment[ptyp] OR Editorial[ptyp] OR Letter[ptyp] OR Case Reports[ptyp] OR News[ptyp] OR Clinical Trial[ptyp]))) NOT (("Animals"[mh] NOT "Humans"[mh]))
