# Supplementary material for: Serious mental health conditions and exposure to adulthood trauma in low- and middle-income countries: a scoping review
Source: Glob Ment Health (Camb). 2024 Nov 20;11:e112. doi: 10.1017/gmh.2024.123 (PMC11704373; doi:10.1017/gmh.2024.123)
Supplement: Peer review history

# Editor report: Recommendation: Serious mental health conditions and exposure to adulthood trauma in low- and middle-income countries: a scoping review — R0/PR2

**DOI:** 10.1017/gmh.2024.123.pr2

**Affiliations:** Centre for Rural Health, University of KwaZulu-Natal, South Africa

In addition to the reviewer comments, please also add a methodological guideline in the methods section, for instance, Arksey and O’Malley, JBI etc.

# Editor report: Decision: Serious mental health conditions and exposure to adulthood trauma in low- and middle-income countries: a scoping review — R0/PR3

**DOI:** 10.1017/gmh.2024.123.pr3

**Affiliations:** London School of Hygiene & Tropical Medicine, United Kingdom of Great Britain and Northern Ireland

No accompanying comment.

# Editor report: Recommendation: Serious mental health conditions and exposure to adulthood trauma in low- and middle-income countries: a scoping review — R1/PR5

**DOI:** 10.1017/gmh.2024.123.pr5

**Affiliations:** Centre for Rural Health, University of KwaZulu-Natal, South Africa

No accompanying comment.

# Editor report: Decision: Serious mental health conditions and exposure to adulthood trauma in low- and middle-income countries: a scoping review — R1/PR6

**DOI:** 10.1017/gmh.2024.123.pr6

**Affiliations:** London School of Hygiene & Tropical Medicine, United Kingdom of Great Britain and Northern Ireland

No accompanying comment.
